# A DNA adenine demethylase impairs PRC2-mediated repression of genes marked by a specific chromatin signature

**DOI:** 10.1186/s13059-023-03042-4

**Published:** 2023-08-30

**Authors:** Qingxiao Jia, Xinran Zhang, Qian Liu, Junjie Li, Wentao Wang, Xuan Ma, Bo Zhu, Sheng Li, Shicheng Gong, Jingjing Tian, Meng Yuan, Yu Zhao, Dao-Xiu Zhou

**Affiliations:** 1 National Key Laboratory of Crop Genetic Improvement, Hubei Hongshan Laboratory, Huazhong Agricultural University, Wuhan, 430070 China; 2 Institute of Plant Science Paris-Saclay (IPS2), CNRS, INRAE, University Paris-Saclay, 91405 Orsay, France

**Keywords:** ALKBH1, DNA adenine methylation (6mA), R-loop, H3K27me3, Polycomb repressive complex 2 (PRC2)

## Abstract

**Background:**

The Fe (II)- and α-ketoglutarate-dependent AlkB family dioxygenases are implicated in nucleotide demethylation. AlkB homolog1 (ALKBH1) is shown to demethylate DNA adenine methylation (6mA) preferentially from single-stranded or unpaired DNA, while its demethylase activity and function in the chromatin context are unclear.

**Results:**

Here, we find that loss-of-function of the rice ALKBH1 gene leads to increased 6mA in the R-loop regions of the genome but has a limited effect on the overall 6mA level. However, in the context of mixed tissues, rather than on individual loci, the ALKBH1 mutation or overexpression mainly affects the expression of genes with a specific combination of chromatin modifications in the body region marked with H3K4me3 and H3K27me3 but depleted of DNA CG methylation. In the similar context of mixed tissues, further analysis reveals that the ALKBH1 protein preferentially binds to genes marked by the chromatin signature and has a function to maintain a high H3K4me3/H3K27me3 ratio by impairing the binding of Polycomb repressive complex 2 (PRC2) to the targets, which is required for both the basal and stress-induced expression of the genes.

**Conclusion:**

Our findings unravel a function of ALKBH1 to control the balance between the antagonistic histone methylations for gene activity and provide insight into the regulatory mechanism of PRC2-mediated H3K27me3 deposition within the gene body region.

**Supplementary Information:**

The online version contains supplementary material available at 10.1186/s13059-023-03042-4.

## Background

N6-adenine methylation (6mA) was first characterized as a DNA modification in bacteria and only identified in a few lower eukaryotes for a long time. More recently, 6mA has been identified in the genomic DNA of a wide range of eukaryotic organisms, from basal fungi to animals and plants, and there exist significant variations in genomic distribution, abundance, and functions of DNA 6mA among eukaryotes [1, 2]. Depending on the organisms, DNA 6mA may function as an epigenetic mark for either transcriptional activation or repression [3–7]. DNA 6mA can be induced during development or under stress. For instance, 6mA is upregulated during the development of mouse trophoblast stem cells, specifically at stress-induced DNA double helix destabilization (SIDD) regions [8]. In rice plants, 6mA was found to be induced by abiotic stress [9]. Deposition of DNA 6mA prevents chromatin binding of SATB1, a crucial chromatin organizer that interacts with SIDD regions, revealing a role of 6mA at the boundaries between euchromatin and heterochromatin to restrict the spread of euchromatin [8]. However, in animals such as *Drosophila*, *C. elegans*, and mammals [5, 10–14], 6mA levels are orders of magnitude lower than those in unicellular eukaryotes such as *Tetrahymena* [15] and *Chlamydomonas* [3]. In higher plants (*Arabidopsis* and rice), DNA 6mA was detected at intermediate levels [4, 7, 9]. Whether low-level 6mA can be accurately detected or even exists in some organisms is currently debated [16–18].

The *E. coli* AlkB protein is an Fe(II)- and alpha-ketoglutarate (α-KG)-dependent dioxygenase that protects the bacterial genome from alkylation damage. AlkB uses molecular oxygen to oxidize the alkyl groups on alkylation-damaged nucleic acid bases, such as 1-methyladenine (m1A) and 3-methylcytosine (m3C) [19, 20]; the oxidized alkyl groups are subsequently released as aldehydes, regenerating the undamaged bases [21]. Proteins homologous to *E. coli* AlkB (ALKBH) have been shown to demethylate 6mA. Human family member ALKBH1, with the highest sequence homology to *E. coli* AlkB, can function as a DNA and RNA demethylase [22, 23] but also exhibits abasic (apyrimidinic/apurinic) lyase activity [24]. Several studies demonstrated that ALKBH1 has the demethylase activity of 6mA on single-stranded DNA [5, 7, 13, 14]. More recent studies indicated that ALKBH1 functions to demethylate 6mA in unpairing regions (e.g., SIDD) of mammalian genomes [25] and promotes SATB1-binding to SIDD regions and euchromatin spreading [8]. ALKBH1 prefers bubbled or bulged DNAs as substrate [25]. Additionally, ALKBH1 can also demethylate histone H2A [26], suggesting that ALKBH1 may have additional functions. Depletion of ALKBH1 led to transcriptional silencing of oncogenic pathways through decreasing chromatin accessibility [14]. The ALKBH1 KO mice showed impaired development phenotypes [27, 28]. Thus, ALKBH1 may have multiple substrates and functions in chromatin and epigenetic regulation.

Histone H3 lysine 27 tri-methylation (H3K27me3) is a repressive chromatin mark deposited by the Polycomb repressive complex 2 (PRC2). In plants, about 10 to 20% of genes are marked by H3K27me3 [29–32], which are mainly involved in developmental processes and stress responses in plants [29, 33, 34]. However, changes in H3K27me3 levels in mutant plants affecting enzymes involved in the H3K27me3 dynamics do not generally correlate with the overall gene expression changes [35–37], suggesting that additional chromatin events may be involved in H3K27me3–mediated gene repression. By contrast, H3K4me3 is an active chromatin mark associated with a large number of expressed genes. Genes marked by both H3K4me3 and H3K27me3 are so-called bivalent genes, which are in many cases poised for activation by cellular stimuli. But how H3K4me3 and H3K27me3 control the expression of bivalent genes is generally unclear.

In this work, we explored the ALKBH1 genome-wide targets and its function in 6mA demethylation, chromatin modifications, and gene expression in plants. Our study uncovered that the ALKBH1-binding antagonizes PRC2 function to maintain a high H3K4me3/H3K27me3 ratio in its target genes to upregulate their expression. These genes are highly enriched in stress response. The results extend the function of ALKBH1 to controlling histone methylation of genes marked by a specific chromatin signature and provide insight into the regulatory mechanism of PRC2 function and H3K27me3 spreading in the gene body.

## Results

### Mutation of ALKBH1 had a limited effect on the genome-wide 6mA in rice

**Fig. 1:**
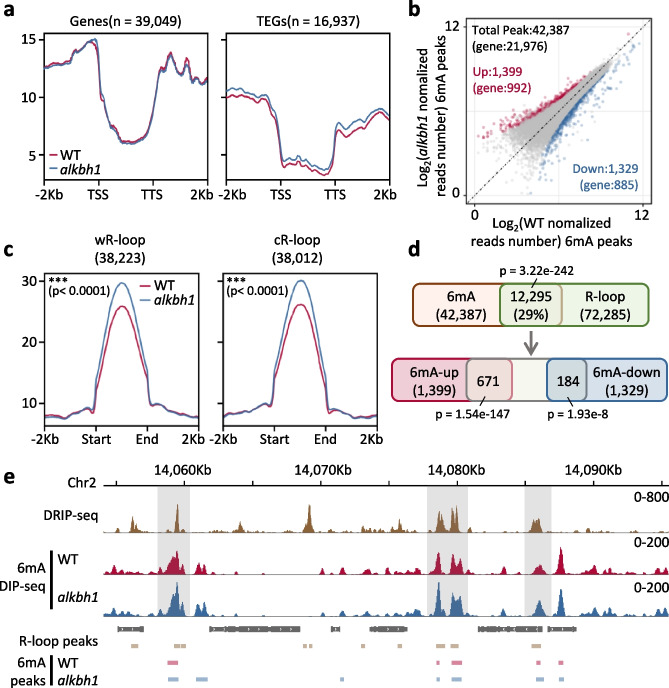
ALKBH1 demethylates 6mA in R-loops in rice. **a** Metaplots of 6mA levels of all genes in *alkbh1* compared with wild type. **b** Scatterplot of 6mA levels between WT and *alkbh1*. DIP-seq peaks merged from all samples (*n* = 42,387) are shown. The *x*-axis and *y*-axis represent the normalized read number at each peak of WT and *alkbh1*, respectively. Blue dots represent the downregulated peaks; red dots represent the upregulated peaks; differential peaks (fold change > 1.5, *P* < 0.05) overlapping between two replicates were considered as significantly changed. **c** Metaplots of 6mA levels at R-loops [39] in *alkbh1* compared with wild type. **d** Venn diagram showing the percentages of 6mA DIP-seq peaks that overlap with DRIP-seq peaks [39] (DNA: RNA hybrid immunoprecipitation and sequencing for R-loops). *P*-values were calculated by hypergeometric tests. **e** DRIP-seq and 6mA DIP-seq signals at a representative genomic region. Gray areas display the difference in signals between the mutant and WT

The rice ALKBH family has 7 members (Additional file 1: Fig. S1a) [7]. The rice ALKBH1 (LOC_Os03g60190) was shown to localize in the nucleus and have an in vitro 6mA demethylase activity toward ssDNA [7]. To study the function of rice ALKBH1 in genome-wide 6mA, we analyzed the wild type (Nipponbare, NIP) and the *alkbh1* CRISPR mutant lines (Additional file 1: Fig. S1b) [7] by using the 6mA DNA immunoprecipitation sequencing (DIP-seq) method [3]. From the DIP-seq, we obtained 32,000 to 37,000 peaks (*P* < 10^−5^) in the wild-type and mutant lines relative to the respective inputs (Additional file 2: Table S1). However, metaplots and scatter plots of the peaks revealed no clear difference in the 6mA levels in genes between the wild type and the mutant, except for some increases in TE-related genes (TEG) in the mutant (Fig. 1a, b). The limited effect of the *alkbh1* mutation on the genome-wide 6mA may be due to a redundant function between ALKBH1 and other members of the family in rice, as a *C. elegans* homolog of ALKBH4 (NMAD-1) was shown to demethylate 6mA [11, 38]. Alternatively, the rice ALKBH1 may target only discrete genomic sites for 6mA demethylation which could not be revealed by the antibody-based DIP-seq method. Previous studies showed that in rice 6mA is enriched in R-loops which are mainly detected in the flanking regions of genes [39]. Analysis of the R-loop 6mA revealed higher levels (*P* < 0.0001) in both Watson DNA strand-related R-loops (wR-loops) and Crick DNA strand-related R-loops (cR-loops) [39, 40] in the mutants than wild type (Fig. 1c), consistent with the results that mammalian ALKBH1 preferentially targets unpaired DNA for 6mA demethylation [25]. In fact, 29% of the 6mA peaks colocalized with R-loops (Fig. 1d). In *alkbh1*, about 48% (671/1399) of the upregulated peaks localized to R-loops compared with 184/1329 downregulated peaks in R-loops (Fig. 1d, e). The analysis suggested a potential role of ALKBH1 to reduce 6mA from R-loops in the rice genome.

### ALKBH1 is required for the expression of genes with a specific chromatin signature

**Fig. 2:**
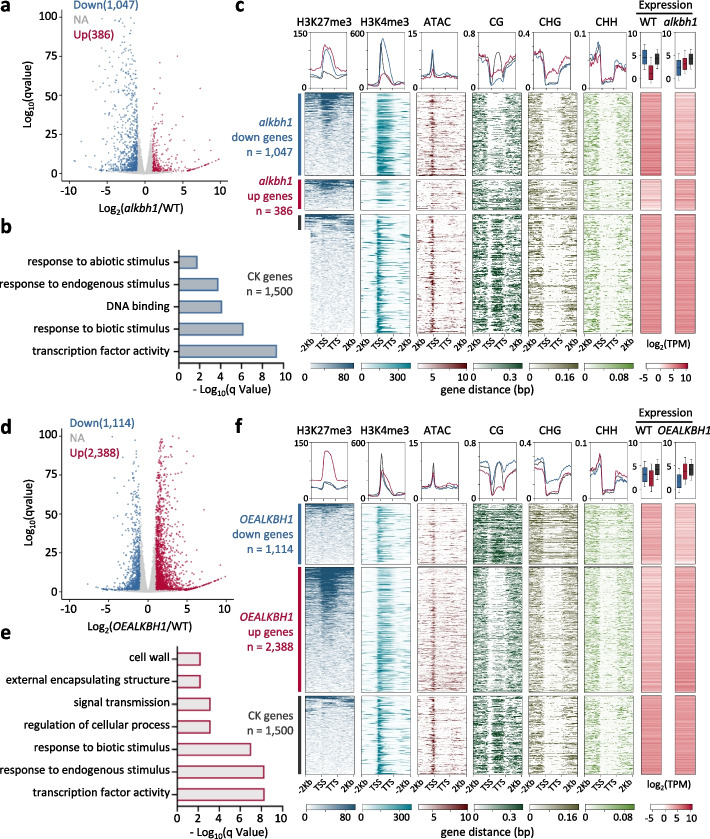
ALKBH1 activates genes marked by both H3K27me3 and H3K4me3 but depleted of mCG in the body region. **a** RNA-seq analysis of differentially expressed genes (DEGs) (> 2-fold; *q*-value < 0.05) in *alkbh1* compared with the wild type (WT). **b** GO enrichment of the downregulated DEGs in *alkbh1*. **c** Metaplots and heatmaps of chromatin modification profiles in the *alkbh1* downregulated (*n* = 1047 blue) and upregulated (*n* = 386 red) genes and control genes with similar transcription levels (CK) (*n* = 1500 gray) in the wild-type background. Expression levels of the DEGs in the mutant and the control genes in wild-type plants are shown by boxplots and heatmaps. **d** RNA-seq analysis of DEGs (> 2-fold; *q*-value < 0.05) in the overexpression lines (*OEALKBH1*) compared with the wild type (WT). **e** GO enrichment of upregulated DEGs in *OEALKBH1*. **f** Metaplots and heatmaps of chromatin modification profiles in the *OEALKBH1* downregulated (*n* = 1114 blue) and upregulated (*n* = 2388 red) genes and control genes (*n* = 1500 gray) in the wild-type background

RNA-seq analysis of seedling tissues uncovered 1047 downregulated and 386 upregulated (> 2 fold, *q* < 0.05) genes in the *alkbh1* mutants compared to the wild-type plants (Fig. 2a), suggesting that ALKBH1 functioned as a positive regulator of gene expression. The downregulated genes were enriched mainly for transcriptional activity and stress response (Fig. 2b), suggesting that ALKBH1 may be a higher hierarchical regulator of basal expression of stress-responsive genes. However, there was no clear association between the gene expression change and the 6mA variation in the mutant (Additional file 1: Fig. S1c). Chromatin modification heatmaps indicated that the downregulated genes had much lower levels of 5′-cytosine methylation (5mC) at CG sequences (mCG) in the body region than the randomly selected 1500 genes (with averaged expression levels) in wild-type background (Fig. 2c). In addition, the downregulated genes exhibited higher levels of H3K27me3 and H3K4me3 in the body region than the control genes in wild-type background. In plants, H3K4me3 mainly peaked at the gene transcriptional start sites (TSS), as observed in the control genes, but the mark was found to be spread all over the body region toward the transcription termination site (TTS) in the downregulated genes (Fig. 2c). By contrast, the upregulated genes in the mutants showed no clear difference in chromatin modifications from the control genes in wild-type background, except for higher levels of H3K27me3 (Fig. 2c).

To confirm the function of ALKBH1 in gene expression, we produced and analyzed the ALKBH1 overexpression (OE) plants that showed no visible growth phenotype (Additional file 1: Fig. S1d, e). RNA-seq analysis detected 2388 up- and 1114 downregulated genes in the *OEALKBH1* plants (Fig. 2d). As the downregulated genes in the mutants, the upregulated genes in the OE plants were also enriched for transcriptional activity and stress response (Fig. 2e) and displayed much higher levels of H3K27me3 and H3K4me3 and lower levels of gene body mCG than the control genes (Fig. 2f). Actually, 265 downregulated genes in the mutants were upregulated in the OE plants (Additional file 1: Fig. S1f, Additional file 3: Table S2). The data indicated that ALKBH1 was required for the expression of genes enriched for H3K27me3 and/or H3K4me3 but without mCG in the gene body in wild-type rice seedling tissues.

Further analysis of the mean transcript levels of genes showing only H3K27me3 or H3K4me3 or both modifications in wild-type seedling mixed tissues indicated that the *alkbh1* mutation and overexpression had more effect on genes marked with H3K27me3 and H3K4me3 than with H3K27me3 or H3K4me3 alone in the context of mixed tissues (Additional file 1: Fig. S1g).

In plants, gene body mCG is associated with gene activity and is detected only in species that express the CHG methyltransferase CMT3 [41, 42]. A correlation between mCG and 6mA in gene body regions was observed [7]. In the rice genome, there were 23,938 genes with (i.e., > 40% methylation) and 7986 genes without (i.e., < 10% methylation) body mCG (Additional file 1: Fig. S2a). The genes without body mCG showed lower 6mA and higher ATAC (open chromatin) signals than those with body mCG (Additional file 1: Fig. S2b). By contrast, these two categories of genes displayed a striking difference in H3K27me3 and H3K4me3: the genes without body mCG showed much higher levels of H3K27me3 and H3K4me3 than those with the body DNA methylation (Additional file 1: Fig. S2b). In addition, the body CG-methylated genes were marked by H3K4me3 only at the TSS, while the body unmethylated genes had H3K4me3 spread from the TSS toward the TTS (Additional file 1: Fig. S2b), reminiscent of the downregulated genes in the *alkbh1* mutants and the upregulated genes in the OE plants (Additional file 1: Fig. S2c). This may reflect the antagonistic relationship between H3K4me3 and CG methylation enzymes [43, 44]. The association of high levels of H3K27me3 and/or H3K4me3 with low levels of DNA methylation in the gene body likely represents a chromatin signature of genes downregulated in *alkbh1* mutant and upregulated in OE plants.

### ALKBH1 binds to genes marked by the specific chromatin signature

**Fig. 3:**
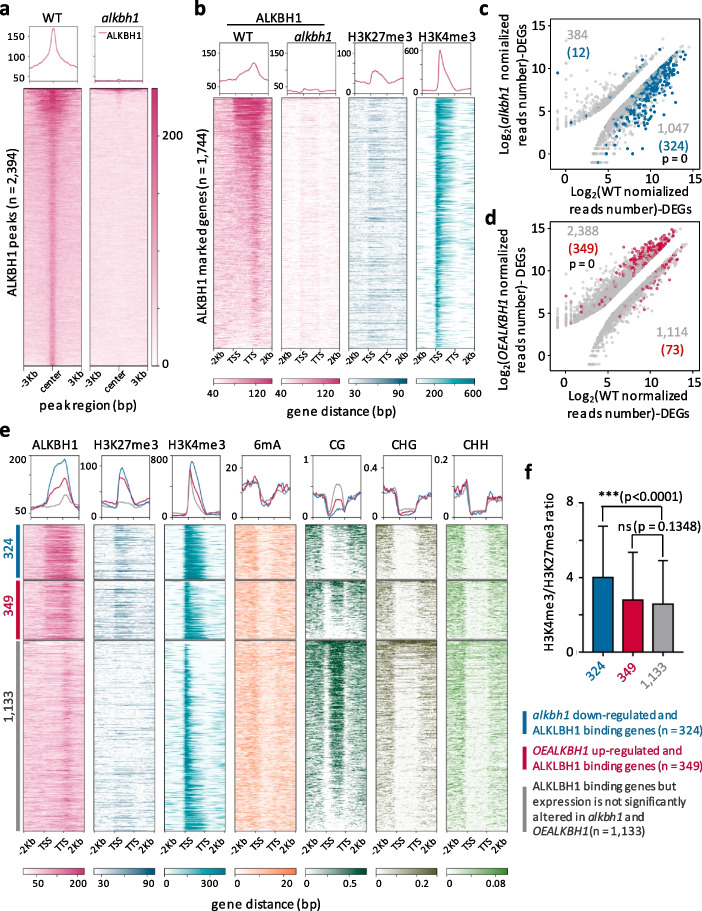
ALKBH1 genome-wide binding characteristics. **a** Metaplots and heatmaps of ALKBH1-binding signals (centered in the binding peaks) in the wild type and *alkbh1#1* mutant. **b** Metaplots and heatmaps of H3K27me3 and H3K4me3 levels in ALKBH1-binding genes. **c** Scatterplots of the differentially expressed genes in *alkbh1* with the ALKBH1-binding genes. The *x*-axis and *y*-axis respectively represent the normalized reads number of the DEGs (log_2_FC > 1, *q*-value < 0.05) in *alkbh1* and in the wild type. The blue dots represent the ALKBH1-binding genes. *P*-values were calculated by hypergeometric tests. **d** Scatterplots of the DEGs in *OEALKBH1* with the ALKBH1-binding genes. The *x*-axis and *y*-axis respectively represent the normalized reads number of the DEGs (log_2_FC > 1, *q*-value < 0.05) in *OEALKBH1* and in the wild type. The red dots represent the ALKBH1-binding genes. *P*-values were calculated by hypergeometric tests. **e** Metaplots and heatmaps of chromatin modification profiles in the ALKBH1-binding genes that are downregulated in *alkbh1* (*n* = 324 blue), upregulated in *OEALKBH1* (*n* = 349 red), and unchanged in the mutant or OE plants (*n* = 1133 gray). **f** Boxplots of H3K4me3/H3K27me3 ratios of the ALKBH1-binding genes that are downregulated in the mutant (324), upregulated in OE plants (349), and unchanged in the transgenic plants (1133). Statistical significance between wild type and *alkbh1* was calculated by Student’s *t*-test. *P < 0.05, **P < 0.01, ***P < 0.001. ns, no significant difference

To further study genomic targets of ALKBH1, we produced an antibody of the protein using a peptide as antigen (Additional file 1: Fig. S3a) and performed ChIP-seq analysis of the wild type and, as a control, the *alkbh1* plants (Additional file 1: Fig. S3b, Additional file 2: Table S1). We detected 2394 ALKBH1-binding peaks (or 1744 binding genes) in the wild type (Fig. 3a). The ALKBH1 binding located mainly in the gene body with the highest binding level toward TTS (Fig. 3b). Heatmap analysis indicated that the ALKBH1 binding levels mirrored that of H3K4me3, whereas the binding genes showed a more or less uniformed level of H3K27me3 (Fig. 3b). Scatter plots revealed that 33% (324/1047, *P*-value = 0) of the downregulated genes in the *alkbh1* mutants and 15% (349/2388, *P*-value = 0) of the upregulated genes in the OE plants were ALKBH1-binding genes (Fig. 3c, d). In these DEGs, the ALKBH1-binding and H3K4me3 levels were higher and the body mCG was much lower than the remaining binding genes (Fig. 3e). These DEGs also showed higher H3K27me3 levels than the remaining binding genes (Fig. 3e). The downregulated DEGs in the mutants displayed a higher H3K4me3/H3K27me3 ratio than the control genes in the wild-type background (Fig. 3f). The analysis confirmed that ALKBH1 targeted genes with the specific combination of chromatin modifications (i.e., high H3K4me3 and H3K27me3 and low body mCG) to stimulate gene expression. In the context of mixed tissues, either a threshold of the ratio between H3K4me3 and H3K27me3 was required for ALKBH1 binding to stimulate gene expression, or the ALKBH1-binding allowed maintaining an H3K4me3/H3K27me3 ratio favorable for gene expression.

### ALKBH1 is required for maintaining a high H3K4me3/H3K27me3 ratio in the target genes

**Fig. 4:**
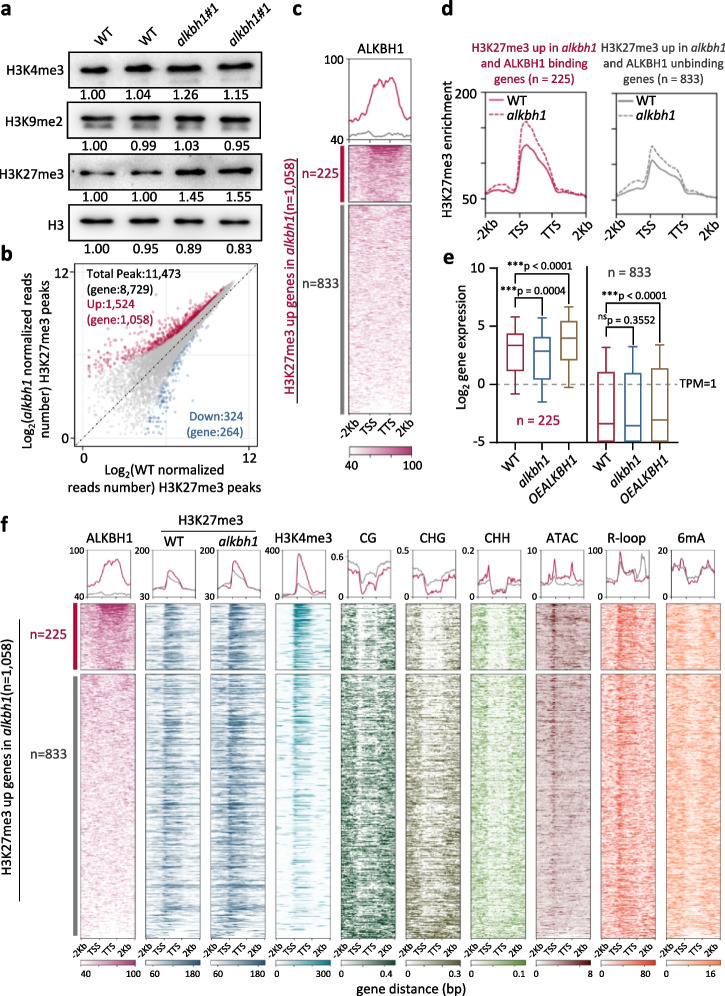
The *alkbh1* mutation increased genome-wide H3K27me3. **a** Histone H3K4me3, H3K9me2, and H3K27me3 levels in *alkbh1* compared to wild types (WT) were detected by immunoblotting. H3 was tested as a loading control. Original images are shown in Additional file 8. **b** Scatterplots of H3K27me3 ChIP-seq peaks in WT and *alkbh1*. Total peaks are merged from all samples (*n* = 11,473). The *x*-axis and *y*-axis represent the normalized read number at each peak of WT and *alkbh1*, respectively. Blue dots represent the downregulated peaks; red dots represent the upregulated peaks; differential H3K27me3 peaks (fold change > 1.5, *P* < 0.05) overlapping between two replicates were considered as significantly changed. **c** Heatmaps and metaplots of ALKBH1-binding signals in the genes that showed increased H3K27me3 in *alkbh1* (*n* = 1058), of which 225 showed ALKBH1 binding, and the remaining (833 gray) are unbound genes. **d** Metaplots of elevated levels of H3K27me3 for ALKBH1-binding and non-binding genes in *alkbh1* versus WT. **e** Boxplots of the expression levels of the two gene categories in **c** in the wild-type, mutant, and OE plants. The *y*-axis represents the gene expression level in log2 (TPM + 0.01). The center line stands for the median, box limits stand for the upper and lower quartiles, and whiskers stand for 1.5 × the interquartile range. The dotted line is TPM equal to 1. Statistical significance between comparisons was calculated by Student’s *t*-test. *P < 0.05, **P < 0.01, ***P < 0.001. ns, no significant difference. **f** Heatmaps and metaplots of ALKBH1 binding and chromatin modifications signals in the genes that showed increased H3K27me3 in *alkbh1* (*n* = 1058). The top 225 genes are ALKBH1-binding genes

**Fig. 5:**
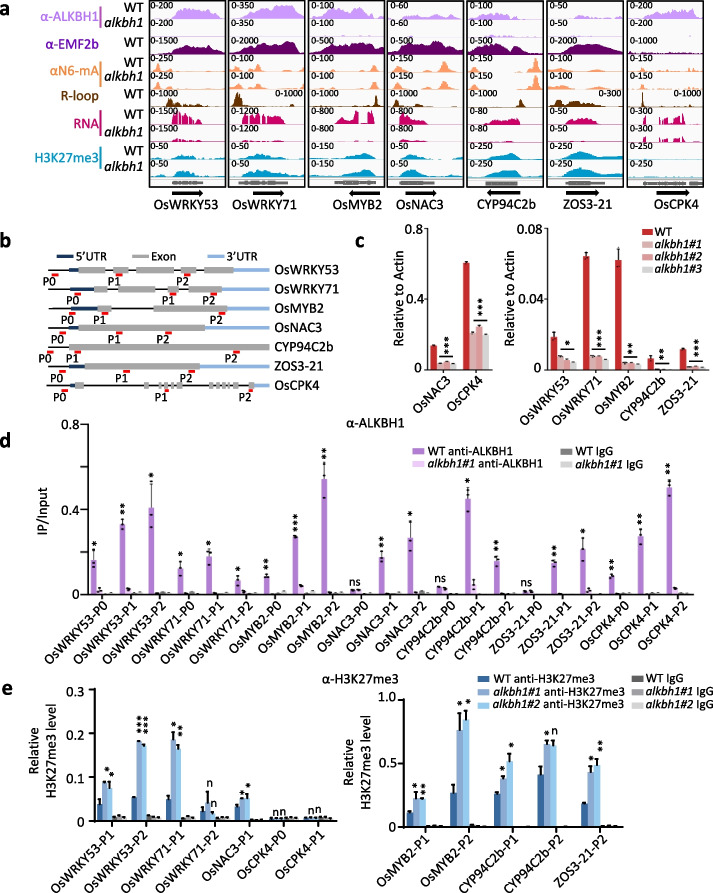
ALKBH1-binding inhibits H3K27me3 and promotes the expression of target genes. **a** Integrative Genomics Viewer of 7 stress-related transcription factor genes targeted by ALKBH1. **b** Three regions (red horizontal bars) of the 7 genes tested by ChIP-PCR analysis with H3K27me3 and ALKBH1 antibodies. **c** RT-PCR analysis of the 7 genes in wild-type and *alkbh1* mutant lines. P2 primers were used. **d** Anti-ALKBH1 ChIP-PCR detection of ALKBH1 association with the 7 genes in wild-type and *alkbh1* plants. **e** ChIP-PCR detection of H3K27me3 levels in the 7 genes in wild-type and *alkbh1* plants. Values are the means of three biological replicates, and error bars represent ± SD. Statistical significance between wild type and *alkbh1* was calculated by Student’s *t*-test. *P < 0.05, **P < 0.01, ***P < 0.001. *n*, no significant difference

To test the above hypotheses, we first analyzed the histone methylation levels in *alkbh1* and wild type plants by immunoblots. The level of H3K27me3, but not H3K4me3 nor H3K9me2, was augmented in the mutants but reduced in the OE plant (Fig. 4a, Additional file 1: Fig. S4a, b). The results suggested that ALKBH1 might modulate H3K27me3 to control the H3K4me3/H3K27me3 ratio on the target genes. Next, we performed H3K27me3 ChIP-seq analysis of the wild type and the *alkbh1* plants and detected a total of 11,473 peaks (or 8729 genes), representing about 14% of the rice genes (Fig. 4b, Additional file 2: Table S1), as found in other studied [29, 35, 37]. In the mutant, 1542 peaks (or 1058 genes) showed an increase but only 324 peaks (264 genes) showed a decrease (fold change > 1.5, *P*-value < 0.05) of H3K27me3 (Fig. 4b), confirming the immunoblot results. Among the 1058 genes, 225 were ALKBH1-binding genes (Additional file 4: Table S3), in which the increases of H3K27me3 in the mutant were more important than remaining 833 genes (Fig. 4c, d) and showed lower expression in the mutants but higher expression in the OE lines than wild type (Fig. 4e), suggesting that ALKBH1-binding reduced H3K27me3 and stimulated gene expression. Interestingly, the 225 ALKBH1-binding genes displayed much higher levels of H3K4me3 than the remaining 833 genes in the wild-type background, while the total 1058 genes with increased H3K27me3 in the mutant had similar H3K27me3 levels in a wild-type background (Fig. 4f). This supported the hypothesis that ALKBH1 preferentially bound to genes marked by a high H3K4me3/H3K27me3 ratio (Fig. 3f) and that ALKBH1 functioned to maintain the ratio by restraining H3K27me3. In addition, the 225 genes showed higher levels of ATAC signals at TSS and TTS and lower levels of 6mA, mCG, and mCHG at gene body and R-loop at TTS in wild-type background (Fig. 4f). Within the 225 genes, 35 were downregulated in the mutant, and 64 were upregulated in the OE plants (Additional file 5: Table S4). More than half (18/35) of the genes encode transcription factors, particularly WRKY, NAC, MYB, and ZOS family members (Fig. 5a, Additional file 5: Table S4), many of which have been shown to regulate stress response [45–47]. The expression, H3K27me3, and ALKBH1-binding of 7 representative genes in wild-type and *alkbh1* plants were validated by RT-PCR and ChIP-PCR (Fig. 5b–e). All of the tested genes were found to be bound by ALKBH1, and the highest binding activity was detected near the TTS (Fig. 5d), confirming the ChIP-seq data. All tested genes showed decreased expression and increased H3K27me3 in the gene body in the mutant, except for CPK4 in which no H3K27me3 was detected (Fig. 5e).

### ALKBH1 inhibits H3K27me3 deposition by preventing PRC2 (EMF2b) binding

**Fig. 6:**
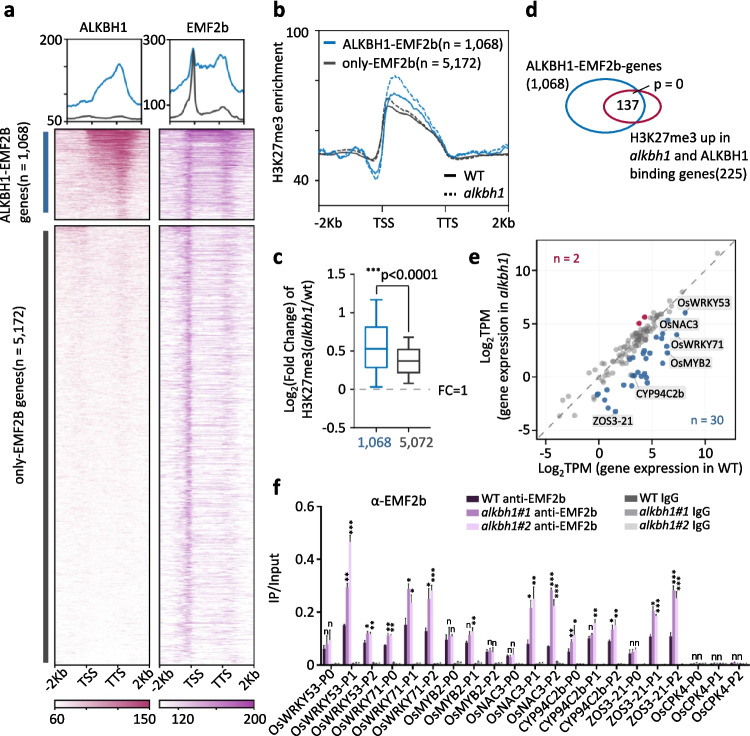
ALKBH1 binds to a subset of the PRC2 (EMF2b) targets in the rice genome. **a** Metaplots and heatmaps of ALKBH1-binding levels in EMF2b binding genes (*n* = 6240). **b** Metaplots of H3K27me3 levels of the genes (1068) commonly bound by EMF2b and ALKBH1 (upper) compared to those bound by EMF2b only (lower) in WT and *alkbh1*. **c** The fold change (log_2_) of H3K27me3 of the two gene populations in *alkbh1* relative to wild type. The *P*-value was calculated based on the *t*-test. **d** The Venn diagram between 1068 genes commonly bound by EMF2b and ALKBH1 and 225 ALKBH1-binding genes that shown increased H3K27me3 in the *alkbh1* mutants. **e** Expression levels (in log_2_ TPM) of the 137 genes in wild type and *alkbh1.* **f** Anti-EMF2b ChIP-PCR detection of EMF2b binding to the 7 ALKBH1-targeted genes in wild type and *alkbh1*. Data represent the means ± SD (three separate experiments per sample). Statistical significance between wild type and *alkbh1* was calculated by Student’s *t*-test. *P < 0.05, **P < 0.01, ***P < 0.001. n, no significant difference

The increases of H3K27me3 in the mutants suggested that ALKBH1 might either remove or inhibit H3K27me3 deposition. A H3K27me3 demethylase activity of ALKBH1 was not excluded, as its catalytic domain is similar to the conserved histone demethylase JmjC domains [21]. In addition, ALKBH1 could demethylate histone H2A [26]. However, tests of ALKBH1 demethylase activity on histones in plant cells turned out to be negative (Additional file 1: Fig. S5). Alternatively, ALKBH1 might prevent PRC2 function to deposit H3K27me3 from its binding targets. To test this hypothesis, we compared the ALKBH1 binding peaks (genes) with those of EMF2b in the rice tissues [37]. EMF2b binds a large number of genomic loci, only part of them showed H3K27me3 [37]. It is suggested those EMF2b-binding sites without H3K27me3 may be pre-nucleation sites for PRC2-mediated H3K27me3 deposition [48, 49]. Actually, more than 60% (1455/2394) of the ALKBH1-binding peaks or genes (1068/1744) were also bound by EMF2b (Fig. 6a), indicating that ALKBH1 mainly associated with the PRC2-binding targets in the rice genome. Interestingly, the ALKBH1-binding genes displayed the highest EMF2b-binding levels especially in the gene body and at TTS regions (Fig. 6a), suggesting that ALKBH1 may preferentially associate with the loci broadly bound by EMF2b. In addition, the genes bound by both ALKBH1 and EMF2b showed higher H3K27me3 levels than those targeted only by EMF2b in the wild type, which was likely due to the fact that a large portion of the EMF2b-binding genes had no H3K27me3 [37]. The *alkbh1* mutation had more effects on H3K27me3 levels of the genes bound by both ALKBH1 and EMF2b than those bound by EMF2b alone (Fig. 6b, c), supporting the hypothesis that ALKBH1 impaired PRC2-mediated H3K27me3 deposition on a subset of PRC2 targets. Among the 255 ALKBH1-binding genes that showed increased H3K27me3 in *alkbh1* mutant, 137 were also bound by EMF2b (Fig. 6d), of which 30 were upregulated and 2 were downregulated in the *alkbh1* mutants (Additional file 6: Table S5, Fig. 6e). The observations suggested that ALKBH1 may either compete with EMF2b for binding to the target genes or impair the EMF2b (or PRC2) function to deposit H3K27me3 from nucleation sites toward gene body to a threshed level that leads to gene repression*.* ChIP-PCR analysis indicated that EMF2b bound 6 of the above analyzed 7 ALKBH1 target genes and the binding levels were largely increased in the gene body of WRKY53, WRKY71, NAC3, and ZOS3 in the *alkbh1* background (Fig. 6f), supporting the assumption that ALKBH1 inhibits the binding of EMF2b in genes with the above-described specific chromatin feature.

To investigate whether 6mA was involved in the ALKBH1 function to control H3K27me3, we analyzed the 6mA peaks that colocalized with R-loops (Fig. 1d). These peaks corresponded to 7484 genes, of which 4425 were marked by H3K27me3 (Additional file 1: Fig. S6a). The marked genes showed higher 6mA, R-loop, H3K4me3, ATAC, and ALKBH1-binding signals than the remaining unmarked 3059 genes that were, by contrast, enriched for TE-related genes (1648/3059), H3K9me2 and DNA methylation (Additional file 1: Fig. S6b), suggesting that ALKBH1 targeted 6mA-methylated R-loops in euchromatin regions. In addition, the H3K27me3 signals of the marked genes displayed a reverse trend of H3K4me3 and ALKBH1 binding signals (Additional file 1: Fig. S6b). The *alkbh1* mutation reduced the expression levels of the 4425 genes but had no effect on the 3059 genes (Additional file 1: Fig. S6c). In the *alkbh1* mutant, 733 genes of the H3K27me3-marked genes showed increased 6mA levels compared with 106 genes with decreases of the modification (Additional file 1: Fig. S6d), and 392 genes showed increased H3K27me3 compared with 78 genes with decreases of the mark (Additional file 1: Fig. S6e). Collectively, the analysis supported the hypothesis that ALKBH1 targeted R-loops within euchromatin to control 6mA and H3K27me3 levels.

### ALKBH1 is required for stress-responsive gene expression and plant tolerance

**Fig. 7:**
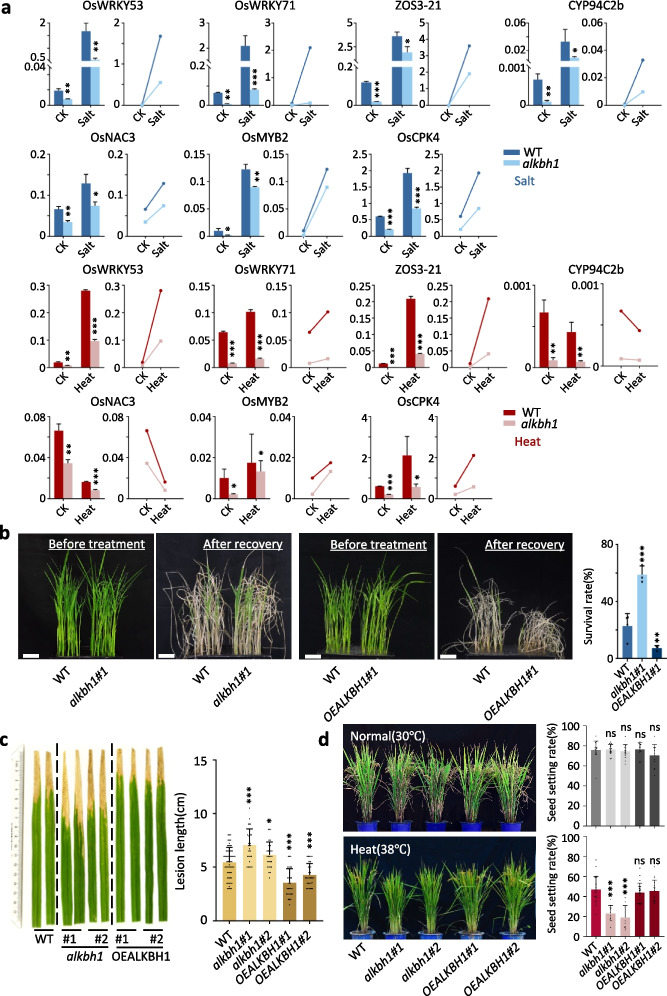
ALKBH1 is required for stress-induced gene expression and is involved in plant tolerance to stress. **a** RT-PCR analysis of ALKBH1 target genes expression in plants treated with salt (green) and high temperature (red). Three biological replicates were performed. Student’s *t*-test was used to detect significance. Significance was calculated by comparing the mutant with the wild type and marked on the mutant column. **b** Recovery phenotypes of wild type and the transgenic plants from salt stress. The seedlings of Nipponbare (NIP, the wild type control with *alkbh1* and *OEALKBH1*), *alkbh1* and *OEALKBH1* grown under 12-h light/12-h dark conditions for 14 days, transferred to 180 mM NaCl for 3 days and recovered for 7 days, respectively. Scale bar, 5 cm. Data are presented as mean ± SD (*n* > 3 biological replicates, and 35 plants were tested in each of the biological replicates). Significance of differences (***P < 0.001) was determined by Student’s *t*-test. Significance was calculated by comparing the mutant or overexpression with the wild type and marked on the mutant and overexpression column. **c** Leaf phenotypes of 8-week-old WT, *alkbh1* and *OEALKBH1* plants inoculated with the *Xoo* isolate PXO99A. Lesion lengths were measured and the values are means ± SEM (*n* > 30 biological replicates). Significance was calculated by comparing the mutant or overexpression with the wild type and marked on the mutant and overexpression column. **d** Phenotype and seed setting rate of the mutant and overexpressed plants under normal and high-temperature conditions. Data are presented as mean ± SD (*n* > 20 biological replicates). Significance of difference (***P < 0.001 was tested by Student’s *t*-test). Significance was calculated by comparing the mutant or overexpression with the wild type and marked on the mutant and overexpression column

Because the downregulated genes in the mutant and the upregulated genes in the OE plants are enriched for stress-responsive function, and many of the 35 genes directly activated by ALKBH1 have been previously shown to function in plant stress responses, we tested stress-induced gene expression and/or tolerance of the transgenic plants to salt, heat, and biotic (infection by *Xanthomonas oryzae* pv. *oryzae*, *Xoo*) stresses. For gene expression, we treated the plants cultured in a hydroponic nutrient solution supplemented with 180 mM NaCl for 12 h or incubated the plants at 42 ° C for 3 h and sampled them for RT-PCR, and the tests revealed that all of the tested genes were induced by the salts and most by the heat stress. Nearly all of the tested genes showed a lower expression in the mutants before and after the stress treatments (Fig. 7a). For plant tolerance experiments, after treatment with NaCl for 3 days, hydroponic cultured plants were transferred to the normal growth medium for 7 days for recovery. The recovery rates of the mutants were actually higher and the OE plants were lower than the wild-type plants (Fig. 7b). Although no clear phenotype was observed during heat stress, the seed setting rate of the mutant was greatly reduced under high temperature (Fig. 7d). By contrast, compared to the wild type, the mutants appeared to be more susceptible and the OE plants were more resistant to the *Xoo* infection (Fig. 7c). The data suggest that ALKBH1-dependent gene expression may have distinct effects in plant response to biotic and abiotic stresses.

## Discussion

### Multiple functions of ALKBH1 in chromatin modification

The present work provides evidence that rice ALKBH1 has a function to restrain H3K27me3 to stimulate the expression of genes with a specific chromatin signature in plants. Although the catalytic domain resembles that of histone demethylase [21], the rice ALKBH1 appears to have no H3K27me3 demethylase activity. The modulation of H3K27me3 by ALKBH1 is likely achieved by inhibiting PRC2 (EMF2b) binding or spreading at the common targets. However, at this stage, it is not excluded that the 6mA demethylase activity of the protein might be also involved, as it was shown in mammalian cells that 6mA deposition preserves genome-wide levels of ubiquitination of H2A lysine 119 (H2Aub) and Polycomb silencing [38]. H2Aub is deposited by PRC1 and recognized by PRC2 to deposit H3K27me3. However, immunoblot analysis did not detect any difference in H2Aub between the wild type and the *alkbh1* mutants or OE plants (Additional file 1: Fig. S4b, c). In addition, 6mA and R-loops were detected in the tested ALKBH1 target genes, but in the regions where the ALKBH1-binding is absent or low (Fig. 5a). This suggests that ALKBH1 may have a function to restrict 6mA spreading and to reduce H3K27me3 in R-loops, which has been shown to enhance PRC2 function [50, 51]. This is supported by the data showing that ALKBH1 preferentially bound to genes enriched for 6mA-methylated R-loops in euchromatin regions and that the *alkbh1* mutation led to increases of both 6mA and H3K27me3 in this gene category (Additional file 1: Fig. S6).

Although no clear variation of H3K4me3 in the *alkbh1* mutants was observed, the ALKBH1-dependent high H3K4me3/H3K27me3 ratio to simulate the expression of the target genes may also involve a function of ALKBH1 to stabilize or recruit H3K4me3 methyltransferases. In Drosophila, the DNA 6mA demethylase DMAD, an ortholog of 5mC demethylase ten-eleven translocation (TET) that is distally related to the AlkB dioxygenase, interacts with the Trithorax-related complex protein Wds (for H3K4me3 deposition) by dynamically demethylating intragenic 6mA [12, 52]. Accumulation of 6mA by depleting DMAD coordinates with Polycomb proteins and contributes to transcriptional repression of these genes [53]. No TET homolog of 5mC demethylase is found in plants so far. The distal relationship of ALKBH1 with TET and its multiple demethylase substrates allow us to speculate that ALKBH1 may have an activity to maintain low mCG levels in the body region of the target genes.

### ALKBH1 targets a specific chromatin signature poised for stress response

In this work, we showed that ALKBH1 preferentially targeted genes marked by the specific chromatin signature (i.e., high H3K4me3 and H3K27me3 but depleted of mCG) in the body region. H3K4me3 is deposited by Trithorax complexes and positively regulates transcription, whereas H3K27me3 is deposited by PRC2 and promotes gene repression [54]. In plants, H3K27me3 is mainly found in the gene body, and its deposition in the Arabidopsis *FLC* gene triggered by cold temperature maintains the repression state of the gene across multiple rounds of cell division during growth [55, 56]. By contrast, at the genomic scale, H3K4me3 is enriched at the TSS site of expressed genes in plants. The present and previous data suggest the two antagonstic histone methylation marks may co-exist in the body region of a set of genes in plants [36], although it is not excluded that the co-marking may be due to cell type heterogeneity of the analyzed tissues. This is reminiscent of the so-called bivalent genes of developmental transcription factors that are lowly expressed in early embryonic cells and are poised for lineage-specific expression during differentiation in mammals. However, the bivalency of the mammalian genes occurs mainly at the promoters or enhancers. In addition, the rice genes marked by H3K4me3 and H3K27me3 are depleted of mCG (Additional file 1: Fig. S2). This is consistent with the observations that PRC2 and H3K4 methyltransferases localize to unmethylated CG islands in the promoter regions in mammalian embryonic cells, and conversely, the presence of H3K27me3 and/or H3K4me3 opposes DNMT activity [57, 58]. In plants, an antagonistic relationship between H3K4me3 and CG methyltransferase MET1 was also established [59]. The two antagonistic histone methylations in the gene body likely mark a transient or intermediate state of gene activity, as those genes are enriched for stress responses in rice plants [36]. This is consistent with the functional enrichments of downregulated genes detected in the *alkbh1* mutants. Our data indicate that ALKBH1 has a function to maintain gene expression levels by modulating the H3K4me3/H3K27me3 ratio. This is supported by the requirement of ALKBH1 for the stress-induced expression of the tested genes and stress tolerance phenotypes of the mutant and OE plants.

## Conclusions

In this work, we discovered that the evolutionarily conserved dioxygenase ALKBH1, previously shown to demethylate 6mA from single-stranded or unpairing DNA, is required to remove 6mA from R-loops in euchromatin regions of the rice genome and has a function to inhibit H3K27me3 or to reduce the H3K27me3/H3K4me3 ratio to promote gene expression. The ALKBH1 function in histone methylations and gene expression essentially relies on impairing PRC2 binding to target genes to deposit the repressive H3K27me3 mark. These results uncover a novel function of ALKBH1 and provide new insights into the regulatory mechanism of PRC2 function to control gene expression.

## Methods

### Rice mutant and overexpression lines

All experiments were performed in the rice (*Oryza sativa spp. japonica*) cultivar Nipponbare (NIP). The *alkbh1#1* and *alkbh1#3* mutants are two previously characterized lines in the NIP background [7]. The *alkbh1#2* mutant was produced using a CRISPR–Cas9 system. CRISPR-P (http://crispr.hzau.edu.cn/CRISPR2/) was used for gRNA design. The sequence GAGGCGAGCCTTCTGCCGCGGGG in ALKBH1 was selected as the specific target. The Gibson Assembly method (*Kpn* I digestion) was used to assemble the gRNA oligonucleotides into the CRISPR system. For the overexpression experiment, the ALKBH1 full-length cDNA without a stop codon was amplified from the NIP cDNA using primers OEALKBH1-F and OEALKBH1-R (Additional file 7: Table S6) and then cloned into the overexpression vector pU1301 in frame with 3XFLAG tag at the 3′ end with *Kpn* I and *BamH* I sites. *alkbh1#1* and *OEALKBH1#1* were used for high-throughput sequencing.

### Plant growth

For high-throughput sequencing, rice seeds were surface sterilized with 75% (v/v) ethanol (2 min) and 0.1% (w/v) HgCl_2_ (12 min) before sowing. Seedlings were grown on half-strength Murashige and Skoog (MS) solid medium (with 0.15% [w/v] phytagel and 2% [w/v] sucrose) in sterilized tubes for 12 days. Plants were grown in a growth chamber under a 16-h light/8-h dark photoperiod with a day temperature between 28 and 32°C and a night temperature between 23 and 26 °C.

### Salt and heat stress treatments

For physiological analysis, seeds were immersed in water at 37 °C for 2 days; then, the germinated seeds were placed into the bottomless 96-well plate that grew in hydroponic nutrient solution [35]. For seedling stress tests, 2-week-old plants were transferred to the hydroponic nutrient solution containing 180 mM NaCl for growing 3 days. Subsequently, they were moved to a normal hydroponic nutrient solution for recovery. After 7 days, the survival rate was calculated according to the percentage of seedlings alive.

For identifying rice thermotolerance at the reproductive stage, the rice plants at the heading stage were transferred from the experimental field to the greenhouse (38 °C, 12 h light/35 °C, 12 h dark) till to maturity [60].

The 2-week-old seedlings were transferred to a hydroponic nutrient solution containing 180 mM NaCl at the seedling stage to grow for 12 h, and then leaf samples were harvested for RNA extraction and RT-PCR. The 2-week-old seedlings were transferred to 42 °C high-temperature incubators for 3 h at the seedling stage, and then leaf samples were harvested for RNA extraction and RT-PCR.

### Pathogen inoculation

Rice flag leaves were inoculated with *X. oryzae* pv. *oryzae* (*Xoo*) strains at the booting (panicle development) stage by the leaf-clipping method as previously described [61]. *Xoo* strains used in this study included Philippine strains *PXO99*. Disease was scored by measuring the lesion length 14 days after inoculation. The bacterial growth rate in rice leaves was determined by counting colony-forming units as described previously [62].

### 6mA DIP-seq and analysis

For 6mA IP-seq, rice genomic DNA was extracted from 2-week-old sterile-cultured seedlings and incubated with RNase A overnight. The 6mA IP-seq was performed using published protocols [3]. Briefly, 5-μg genomic DNA was sonicated into 200–400 bp and end-repaired, followed by adapter ligation. DNA was denatured at 95 °C for 10min and chilled on ice for 10 min. An aliquot of the denatured DNA was saved as input. The remaining DNA was immunoprecipitated overnight at 4 °C with anti-6mA antibodies purchased from SYSY (202-003). DNA was eluted by 6mA salt competition. After size selection and PCR amplification, the DNA fragments were sequenced with the HiSeq 2500 (Illumina). Low-quality reads were removed from raw data by using the Trimmomatic package (http://www.usadellab.org/cms/uploads/supplementary/Trimmomatic/Trimmomatic-Src-0.35.zip), and the clean data were mapped to the MSU7.0 rice reference genome (ftp://ftp.plantbiology.msu.edu/pub/data/Eukaryotic_Projects/o_sativa/annotation_dbs/pseudomolecules/version_7.0/all.dir/) using Bowtie2 with parameters permitting less than two mismatches. The reads were mapped to bacterial, fungal, and archaeal genomes (ftp://ftp.ncbi.nlm.nih.gov/genomes/genbank/). Then, MACS (model-based analysis of chromatin immunoprecipitation followed by sequencing (ChIP-seq) [63]) was introduced to locate enriched regions to call 6mA peaks by comparing reads from the IP sample with the input sample. The DiffBind (version 3.13) Bioconductor package [64] was used to find differential peaks between *alkbh1* and WT samples. The DESeq2 method implemented in DiffBind was used to test the differential peaks. Fold change > 1.5 and *P*-value < 0.05 were used to define differential binding peaks.

### ALKBH1 antibody preparation

Preparation of polyclonal antibodies in rabbits using rice ALKBH1-specific peptide (amino acid [aa] 21–38) as antigen. The peptide (C-RQKARLPRGPVHEKSLEQ) (24mg) was synthesized in vitro. After three cycles of injections, the antisera were obtained, affinity-purified using antigen-coupled magnetic beads, and mixed with 20% glycerol for storage at − 80 °C.

### RNA-seq and data analysis

Total RNA of 2-week-old rice seedling was isolated with TRIzol Reagent (Invitrogen). The RNA-seq libraries were prepared using the Illumina TruSeq RNA Sample Preparation Kit and sequenced on an Illumina HiSeq 2500 instrument (paired-end, 2 × 150bp). RNA-seq data were filtered by Trimmomatic (version 0.33) to remove reads that failed to align to the rice genome and low-quality reads. Hisat2 (version 2.1.0) [65] was used to map the clean reads to the rice reference genome (MSU 7.0, http://rice.plantbiology.msu.edu/). FeatureCounts (v2.0.0) [66] and DESeq2 (v1.26.0) [67] were used to calculate DEGs with an adjusted FDR < 0.05 and fold change > 2.

### ChIP

Two grams of 2-week-old rice seedling was cross-linked by 1% (v/v) formaldehyde and used for chromatin extraction. Chromatin was fragmented to around 200 bp by sonication and then incubated with antibody-coated beads (Invitrogen/Life Technologies; 10001D) overnight. After extensive washing, immunoprecipitated chromatin was de-cross-linked and retrieved, and unimmunoprecipitated chromatin was used as input. The precipitated and input DNA samples were analyzed by high-throughput sequencing or by RT-PCR with gene-specific primers listed in (Additional file 7: Table S6). Anti-H3K27me3 (Millipore; 07-449), anti-ALKBH1 (this work), anti-EMF2b (Tan et al 2022), and anti-IgG (Abcam; ab37415) antibodies were used.

### ChIP-seq data analysis

DNA from chromatin immunoprecipitation was used to construct sequencing libraries following the protocol provided by the Illumina TruSeq ChIP Sample Prep Set A and sequenced on Illumina HiSeq 2500. Trimmomatic (version 0.32) was used to filter out low-quality reads. Clean reads were mapped to the rice genome (MSU7.0; http://rice.plantbiology.msu.edu/) by Bowtie2 (version 2.4.2) using default parameters. SAMtools (version 1.9) was used to remove potential PCR duplicates. The bam files were first converted to Wiggle files, and bigwig files were generated using bamCoverage with the parameters “-bs 10 -normalizeUsing RPKM” in DeepTools [68], and the data were imported into the Integrated Genome Browser for visualization. The two replicates were merged using MACS2 in peak calling (version 2.1.1) [63], for H3K27me3 and ALKBH1 with the parameters “--broad --broad-cutoff 0.1”. The reads from input were used as controls for H3K27me3 and ALKBH1 ChIP-seq, respectively. The DiffBind (version 3.13) Bioconductor package [64] was used to find differential peaks between *alkbh1* and WT samples. The DESeq2 method implemented in DiffBind was used to test the differential peaks. fold change > 1.5 and *P*-value < 0.05 were used to define differential binding peaks.

### Protein extraction and western blot

Histones were extracted using The EpiQuik™ Total Histone Extraction Kit by following the manufacturer’s instruction (Epigentek; OP-0006-100) and then detected by western blot with anti-H3 (Abcam; ab1791), anti-H3K27me3 (Millipore; 07-449), anti-H3K9me2 (Abcam; ab1220), and anti-H3K4me3 (Abcam; ab8580).

### Reverse transcription and RT-PCR

One microgram of total RNA was reverse-transcribed in a reaction volume of 20 μL using DNase and reverse transcriptase (Vazyme; Nanjing, China; R233-01) according to the manufacturer’s instructions to obtain cDNA. RT-PCR was performed as follows: 95 °C for 10 s, 45 cycles of 95 °C for 5 s, and 60 °C for 40 s. Disassociation curve analysis was performed at 95 °C for 15 s, 60 °C for 20 s, and 95 °C for 15 s. Three biological replicates were performed (separate experiments), each with three technical repeats (three identical samples within an experiment). Data were collected using the ABI PRISM 7500 sequence detection system. The rice ACTIN1 gene was used as the internal control. The primers used for RT-PCR are listed in (Additional file 7: Table S6).

### Immunostaining assays

The H3K27me3 demethylase activity of ALKBH1 was tested in vivo by immunostaining assays. *Nicotiana benthamiana* plants were transiently infiltrated with the 35Spro:ALKBH1-2xHA-2xFLAG or 35Spro:JMJ705-2xHA-2xFLAG (as positive control) construct and grown for 3 days before nuclei isolation by flow cytometry (BD FACSAria III) [35]. Isolated nuclei were visualized by DAPI staining (blue), and the accumulation of the HA fusion protein was revealed by anti-HA immunostaining (Sigma; H3663, 1:100 dilution, green) and histone methylation levels by anti-H3K27me3 immunostaining (Millipore; 07449, 1:100 dilution, red).

### Phylogenetic analysis

The ALKB sequences of *E. coli*, mouse, human, and *Arabidopsis* were downloaded from published data and used for alignment with rice sequences [5, 13, 69]. The ClustalX program was used to generate alignments of ALKB protein sequences. The phylogenetic tree generation was performed by MEGA7.

### Accession numbers

Sequence data from this article can be found in the GenBank/EMBL libraries under the following accession numbers: *OsALKBH1* (LOC_Os03g60190), *EMF2b* (LOC_Os09g13630), *OsWRKY53* (LOC_Os05g27730), *OsWRKY71* (LOC_Os02g08440), *OsMYB2* (LOC_Os03g20090), *OsNAC3* (LOC_Os07g12340), *CYP94C2b* (LOC_Os12g05440), *ZOS3-21* (LOC_Os03g60560), *OsCPK4* (LOC_Os02g03410), *OsALKBH1A* (LOC_Os11g29690), *OsALKBH2*(LOC_Os06g17830), *OsALKBH5* (LOC_Os06g04660), *OsALKBH8A* (LOC_Os04g51360), *OsALKBH8B* (LOC_Os11g43610), *OsALKBH10* (LOC_Os05g33310). The accession numbers of the genes are as follows: *HsALKBH1* (Q13686), *HsALKBH4* (Q9NXW9), *MmALKBH1* (P0CB42), and *CeNMAD-1* (Q8MNT9).

### Supplementary Information

**Additional file 1:** **Fig. S1.** Production and analysis of ALKBH1 mutants and Overexpression lines. **Fig. S2.** A set of genes display a specific chromatin signature in the rice genome. **Fig. S3.** Production and test of ALKBH1 antibody for ChIP-seq. **Fig. S4.** Histone modifications levels in ALKBH1 mutant and over-expression plants.** Fig. S5.** Tests of ALKBH1 H3K27me3 demethylase activity. **Fig. S6.** ALKBH1 targets to 6mA-methylated R-loop containing genes with euchromatin features to reduced 6mA and H3K27me3 levels.

**Additional file 2:** **Table S1.** 6mA DIP-seq, RNA-seq and ChIP-seq data alignment summary.

**Additional file 3:** **Table S2.** 265 genes downregulated in *alkbh1* mutants while upregulated in overexpression.

**Additional file 4:** **Table S3.** ALKBH1 binding and increased enrichment of H3K27me3 in mutants with 225 genes.

**Additional file 5:** **Table S4.** The increases of H3K27me3, expression level downregulated in *alkbh1* and ALKBH1 binding genes.

**Additional file 6:** **Table S5.** ALKBH1-binding genes with H3K27me3 increased in *alkbh1* mutant were also bound by EMF2b，of which 30 genes were upregulated in the *alkbh1*.

**Additional file 7:** **Table S6.** Primers used in this study.

**Additional file 8.** Uncropped images for the blots.

**Additional file 9.** Review history.

## Data Availability

The RNA-seq, 6mA-IP-seq, and ChIP-seq data described in this paper have been deposited Gene Expression Omnibus database under NCBI with the accession number GSE222734 [70]. Publicly available data sets used in this study are as follows: GSE111944 (R-loop) [71], GSE142570 (H3K4me3) [72], GSE128434 (ATAC) [73], GSE81436 (5mC) [74], and PRJNA758693 (anti-EMF2b) [75]. No other scripts and software were used other than those mentioned in the “ Methods” section.

## References

[1] Boulias K, Greer EL (2022). Means, mechanisms and consequences of adenine methylation in DNA. Nat Rev Genet.

[2] Luo GZ, He C (2017). DNA N^6^-methyladenine in metazoans: functional epigenetic mark or bystander?. Nat Struct Mol Biol.

[3] Fu Y, Luo GZ, Chen K, Deng X, Yu M, Han D, Hao Z, Liu J, Lu X, Dore LC (2015). N^6^-methyldeoxyadenosine marks active transcription start sites in Chlamydomonas. Cell.

[4] Liang Z, Shen L, Cui X, Bao S, Geng Y, Yu G, Liang F, Xie S, Lu T, Gu X, Yu H (2018). DNA N^6^-adenine methylation in Arabidopsis thaliana. Dev Cell.

[5] Wu TP, Wang T, Seetin MG, Lai Y, Zhu S, Lin K, Liu Y, Byrum SD, Mackintosh SG, Zhong M (2016). DNA methylation on N(6)-adenine in mammalian embryonic stem cells. Nature.

[6] Wang X, Li Z, Zhang Q, Li B, Lu C, Li W, Cheng T, Xia Q, Zhao P. DNA methylation on N6-adenine in lepidopteran Bombyx mori. Biochim Biophys Acta Gene Regul Mech. 2018;S1874-9399(18)30215–3. 10.1016/j.bbagrm.2018.07.013 30071347

[7] Zhou C, Wang C, Liu H, Zhou Q, Liu Q, Guo Y, Peng T, Song J, Zhang J, Chen L (2018). Identification and analysis of adenine N(6)-methylation sites in the rice genome. Nat Plants.

[8] Li Z, Zhao S, Nelakanti RV, Lin K, Wu TP, Alderman MH, Guo C, Wang P, Zhang M, Min W (2020). N^6^-methyladenine in DNA antagonizes SATB1 in early development. Nature.

[9] Zhang Q, Liang Z, Cui X, Ji C, Li Y, Zhang P, Liu J, Riaz A, Yao P, Liu M (2018). N^6^-methyladenine DNA methylation in Japonica and Indica rice genomes and its association with gene expression, plant development, and stress responses. Mol Plant.

[10] Liu J, Zhu Y, Luo GZ, Wang X, Yue Y, Wang X, Zong X, Chen K, Yin H, Fu Y (2016). Abundant DNA 6mA methylation during early embryogenesis of zebrafish and pig. Nat Commun.

[11] Greer EL, Blanco MA, Gu L, Sendinc E, Liu J, Aristizabal-Corrales D, Hsu CH, Aravind L, He C, Shi Y (2015). DNA methylation on N6-adenine in C. elegans. Cell.

[12] Zhang G, Huang H, Liu D, Cheng Y, Liu X, Zhang W, Yin R, Zhang D, Zhang P, Liu J (2015). N6-methyladenine DNA modification in Drosophila. Cell.

[13] Xiao CL, Zhu S, He M, Chen D, Zhang Q, Chen Y, Yu G, Liu J, Xie SQ, Luo F (2018). N^6^-methyladenine DNA modification in the human genome. Mol Cell.

[14] Xie Q, Wu TP, Gimple RC, Li Z, Prager BC, Wu Q, Yu Y, Wang P, Wang Y, Gorkin DU (2018). N^6^-methyladenine DNA modification in glioblastoma. Cell.

[15] Wang Y, Chen X, Sheng Y, Liu Y, Gao S (2017). N6-adenine DNA methylation is associated with the linker DNA of H2A.Z-containing well-positioned nucleosomes in Pol II-transcribed genes in Tetrahymena. Nucleic Acids Res.

[16] Douvlataniotis K, Bensberg M, Lentini A, Gylemo B, Nestor CE (2020). No evidence for DNA N^6^-methyladenine in mammals. Sci Adv.

[17] Kong Y, Cao L, Deikus G, Fan Y, Mead EA, Lai W, Zhang Y, Yong R, Sebra R, Wang H (2022). Critical assessment of DNA adenine methylation in eukaryotes using quantitative deconvolution. Science.

[18] Schiffers S, Ebert C, Rahimoff R, Kosmatchev O, Steinbacher J, Bohne AV, Spada F, Michalakis S, Nickelsen J, Muller M, Carell T (2017). Quantitative LC-MS provides no evidence for m^6^dA or m^4^dC in the genome of mouse embryonic stem cells and tissues. Angew Chem Int Ed Engl.

[19] Trewick SC, Henshaw TF, Hausinger RP, Lindahl T, Sedgwick B (2002). Oxidative demethylation by Escherichia coli AlkB directly reverts DNA base damage. Nature.

[20] Falnes PO, Johansen RF, Seeberg E (2002). AlkB-mediated oxidative demethylation reverses DNA damage in Escherichia coli. Nature.

[21] Fedeles BI, Singh V, Delaney JC, Li D, Essigmann JM (2015). The AlkB family of Fe(II)/alpha-Ketoglutarate-dependent dioxygenases: repairing nucleic acid alkylation damage and beyond. J Biol Chem.

[22] Westbye MP, Feyzi E, Aas PA, Vagbo CB, Talstad VA, Kavli B, Hagen L, Sundheim O, Akbari M, Liabakk NB (2008). Human AlkB homolog 1 is a mitochondrial protein that demethylates 3-methylcytosine in DNA and RNA. J Biol Chem.

[23] Liu F, Clark W, Luo G, Wang X, Fu Y, Wei J, Wang X, Hao Z, Dai Q, Zheng G (2016). ALKBH1-mediated tRNA demethylation regulates translation. Cell.

[24] Muller TA, Andrzejak MM, Hausinger RP (2013). A covalent protein-DNA 5’-product adduct is generated following AP lyase activity of human ALKBH1 (AlkB homologue 1). Biochem J.

[25] Zhang M, Yang S, Nelakanti R, Zhao W, Liu G, Li Z, Liu X, Wu T, Xiao A, Li H (2020). Mammalian ALKBH1 serves as an N^6^-mA demethylase of unpairing DNA. Cell Res.

[26] Ougland R, Lando D, Jonson I, Dahl JA, Moen MN, Nordstrand LM, Rognes T, Lee JT, Klungland A, Kouzarides T, Larsen E (2012). ALKBH1 is a histone H2A dioxygenase involved in neural differentiation. Stem Cells.

[27] Pan Z, Sikandar S, Witherspoon M, Dizon D, Nguyen T, Benirschke K, Wiley C, Vrana P, Lipkin SM (2008). Impaired placental trophoblast lineage differentiation in Alkbh1^-/-^ mice. Dev Dyn.

[28] Nordstrand LM, Svard J, Larsen E, Nilsen A, Ougland R, Furu K, Lien GF, Rognes T, Namekawa SH, Lee JT, Klungland A (2010). Mice lacking Alkbh1 display sex-ratio distortion and unilateral eye defects. PLoS One.

[29] Liu X, Zhou S, Wang W, Ye Y, Zhao Y, Xu Q, Zhou C, Tan F, Cheng S, Zhou DX (2015). Regulation of histone methylation and reprogramming of gene expression in the rice inflorescence meristem. Plant Cell.

[30] He G, Zhu X, Elling AA, Chen L, Wang X, Guo L, Liang M, He H, Zhang H, Chen F (2010). Global epigenetic and transcriptional trends among two rice subspecies and their reciprocal hybrids. Plant Cell.

[31] Hu Y, Liu D, Zhong X, Zhang C, Zhang Q, Zhou DX (2012). CHD3 protein recognizes and regulates methylated histone H3 lysines 4 and 27 over a subset of targets in the rice genome. Proc Natl Acad Sci U S A.

[32] Turck F, Roudier F, Farrona S, Martin-Magniette ML, Guillaume E, Buisine N, Gagnot S, Martienssen RA, Coupland G, Colot V (2007). Arabidopsis TFL2/LHP1 specifically associates with genes marked by trimethylation of histone H3 lysine 27. PLoS Genet.

[33] Zheng B, Chen X (2011). Dynamics of histone H3 lysine 27 trimethylation in plant development. Curr Opin Plant Biol.

[34] Li T, Chen X, Zhong X, Zhao Y, Liu X, Zhou S, Cheng S, Zhou DX (2013). Jumonji C domain protein JMJ705-mediated removal of histone H3 lysine 27 trimethylation is involved in defense-related gene activation in rice. Plant Cell..

[35] Wang W, Lu Y, Li J, Zhang X, Hu F, Zhao Y, Zhou DX (2021). SnRK1 stimulates the histone H3K27me3 demethylase JMJ705 to regulate a transcriptional switch to control energy homeostasis. Plant Cell.

[36] Lu Y, Tan F, Zhao Y, Zhou S, Chen X, Hu Y, Zhou DX (2020). A chromodomain-helicase-DNA-binding factor functions in chromatin modification and gene regulation. Plant Physiol.

[37] Tan FQ, Wang W, Li J, Lu Y, Zhu B, Hu F, Li Q, Zhao Y, Zhou DX (2022). A coiled-coil protein associates Polycomb repressive complex 2 with KNOX/BELL transcription factors to maintain silencing of cell differentiation-promoting genes in the shoot apex. Plant Cell.

[38] Kweon SM, Chen Y, Moon E, Kvederaviciute K, Klimasauskas S, Feldman DE (2019). An Adversarial DNA N^6^-methyladenine-sensor network preserves Polycomb silencing. Mol Cell.

[39] Fang Y, Chen L, Lin K, Feng Y, Zhang P, Pan X, Sanders J, Wu Y, Wang XE, Su Z (2019). Characterization of functional relationships of R-loops with gene transcription and epigenetic modifications in rice. Genome Res.

[40] Xu W, Xu H, Li K, Fan Y, Liu Y, Yang X, Sun Q (2017). The R-loop is a common chromatin feature of the Arabidopsis genome. Nat Plants.

[41] Bewick AJ, Ji L, Niederhuth CE, Willing EM, Hofmeister BT, Shi X, Wang L, Lu Z, Rohr NA, Hartwig B (2016). On the origin and evolutionary consequences of gene body DNA methylation. Proc Natl Acad Sci U S A.

[42] Bewick AJ, Niederhuth CE, Ji L, Rohr NA, Griffin PT, Leebens-Mack J, Schmitz RJ (2017). The evolution of CHROMOMETHYLASES and gene body DNA methylation in plants. Genome Biol.

[43] Ooi SK, Qiu C, Bernstein E, Li K, Jia D, Yang Z, Erdjument-Bromage H, Tempst P, Lin SP, Allis CD (2007). DNMT3L connects unmethylated lysine 4 of histone H3 to de novo methylation of DNA. Nature.

[44] Greenberg MVC, Deleris A, Hale CJ, Liu A, Feng SH, Jacobsen SE. Interplay between active chromatin marks and RNA-directed DNA methylation in Arabidopsis thaliana. Plos Genet*.* 2013;9(11):e1003946. 10.1371/journal.pgen.1003946 PMC3820799 24244201

[45] Liu X, Bai X, Wang X, Chu C (2007). OsWRKY71, a rice transcription factor, is involved in rice defense response. J Plant Physiol.

[46] Huang J, Sun S, Xu D, Lan H, Sun H, Wang Z, Bao Y, Wang J, Tang H, Zhang H (2012). A TFIIIA-type zinc finger protein confers multiple abiotic stress tolerances in transgenic rice (Oryza sativa L.). Plant Mol Biol..

[47] Xie W, Ke Y, Cao J, Wang S, Yuan M (2021). Knock out of transcription factor WRKY53 thickens sclerenchyma cell walls, confers bacterial blight resistance. Plant Physiol.

[48] Xiao J, Jin R, Yu X, Shen M, Wagner JD, Pai A, Song C, Zhuang M, Klasfeld S, He C (2017). Cis and trans determinants of epigenetic silencing by Polycomb repressive complex 2 in Arabidopsis. Nat Genet.

[49] Laugesen A, Hojfeldt JW, Helin K (2019). Molecular mechanisms directing PRC2 recruitment and H3K27 methylation. Mol Cell.

[50] Skourti-Stathaki K, Torlai Triglia E, Warburton M, Voigt P, Bird A, Pombo A (2019). R-loops enhance Polycomb repression at a subset of developmental regulator genes. Mol Cell.

[51] Alecki C, Chiwara V, Sanz LA, Grau D, Arias Perez O, Boulier EL, Armache KJ, Chedin F, Francis NJ (2020). RNA-DNA strand exchange by the Drosophila Polycomb complex PRC2. Nat Commun.

[52] Hill PW, Amouroux R, Hajkova P (2014). DNA demethylation, Tet proteins and 5-hydroxymethylcytosine in epigenetic reprogramming: an emerging complex story. Genomics.

[53] Yao B, Li Y, Wang Z, Chen L, Poidevin M, Zhang C, Lin L, Wang F, Bao H, Jiao B (2018). Active N^6^-methyladenine demethylation by DMAD regulates gene expression by coordinating with Polycomb protein in neurons. Mol Cell.

[54] Schuettengruber B, Bourbon HM, Di Croce L, Cavalli G (2017). Genome Regulation by Polycomb and Trithorax: 70 years and counting. Cell.

[55] Kim DH, Xi Y, Sung S (2017). Modular function of long noncoding RNA, COLDAIR, in the vernalization response. PLoS Genet.

[56] Buzas DM, Robertson M, Finnegan EJ, Helliwell CA (2011). Transcription-dependence of histone H3 lysine 27 trimethylation at the Arabidopsis polycomb target gene FLC. Plant J.

[57] Ku M, Koche RP, Rheinbay E, Mendenhall EM, Endoh M, Mikkelsen TS, Presser A, Nusbaum C, Xie X, Chi AS (2008). Genomewide analysis of PRC1 and PRC2 occupancy identifies two classes of bivalent domains. PLoS Genet.

[58] Clouaire T, Webb S, Skene P, Illingworth R, Kerr A, Andrews R, Lee JH, Skalnik D, Bird A (2012). Cfp1 integrates both CpG content and gene activity for accurate H3K4me3 deposition in embryonic stem cells. Genes Dev.

[59] Zhang X, Bernatavichute YV, Cokus S, Pellegrini M, Jacobsen SE (2009). Genome-wide analysis of mono-, di- and trimethylation of histone H3 lysine 4 in Arabidopsis thaliana. Genome Biol.

[60] Li XM, Chao DY, Wu Y, Huang XH, Chen K, Cui LG, Su L, Ye WW, Chen H, Chen HC (2015). Natural alleles of a proteasome alpha 2 subunit gene contribute to thermotolerance and adaptation of African rice. Nat Genet.

[61] Hui S, Liu H, Zhang M, Chen D, Li Q, Tian J, Xiao J, Li X, Wang S, Yuan M (2019). The host basal transcription factor IIA subunits coordinate for facilitating infection of TALEs-carrying bacterial pathogens in rice. Plant Sci.

[62] Yuan M, Ke Y, Huang R, Ma L, Yang Z, Chu Z, Xiao J, Li X, Wang S. A host basal transcription factor is a key component for infection of rice by TALE-carrying bacteria. Elife*.* 2016;5:e19605. 10.7554/eLife.19605 PMC4993585 27472897

[63] Zhang Y, Liu T, Meyer CA, Eeckhoute J, Johnson DS, Bernstein BE, Nusbaum C, Myers RM, Brown M, Li W, Liu XS (2008). Model-based analysis of ChIP-Seq (MACS). Genome Biol.

[64] Ross-Innes CS, Stark R, Teschendorff AE, Holmes KA, Ali HR, Dunning MJ, Brown GD, Gojis O, Ellis IO, Green AR (2012). Differential oestrogen receptor binding is associated with clinical outcome in breast cancer. Nature.

[65] Kim D, Paggi JM, Park C, Bennett C, Salzberg SL (2019). Graph-based genome alignment and genotyping with HISAT2 and HISAT-genotype. Nat Biotechnol.

[66] Liao Y, Smyth GK, Shi W (2014). featureCounts: an efficient general purpose program for assigning sequence reads to genomic features. Bioinformatics.

[67] Love MI, Huber W, Anders S (2014). Moderated estimation of fold change and dispersion for RNA-seq data with DESeq2. Genome Biol.

[68] Ramirez F, Dundar F, Diehl S, Gruning BA, Manke T (2014). deepTools: a flexible platform for exploring deep-sequencing data. Nucleic Acids Res.

[69] Mielecki D, Zugaj DL, Muszewska A, Piwowarski J, Chojnacka A, Mielecki M, Nieminuszczy J, Grynberg M, Grzesiuk E (2012). Novel AlkB dioxygenases–alternative models for in silico and in vivo studies. PLoS One.

[70] Jia Q, Zhang X, Liu Q, Li J, WANG W, Ma X, Zhu B, Li S, Gong S, Tian J, et al. A DNA adenine demethylase impairs PRC2-mediated repression of genes marked by a specific chromatin signature.GSE222734. Gene Expression Omnibus. 2023 . https://www.ncbi.nlm.nih.gov/geo/query/acc.cgi?acc=GSE222734. 10.1186/s13059-023-03042-4 PMC10469495 37649077

[71] Fang Y, Chen L, Lin K, Feng Y, Zhang P, Pan X, Sanders J, Wu Y, Wang XE, Su Z, et al. Characterization of functional relationships of R-loops with gene transcription and epigenetic modifications in rice.GSE111944. Gene Expression Omnibus. 2019. https://www.ncbi.nlm.nih.gov/geo/query/acc.cgi?acc=GSE111944. 10.1101/gr.246009.118 PMC6673715 31262943

[72] Zhao L, Xie L, Zhang Q, Ouyang W, Deng L, Guan P, Ma M, Li Y, Zhang Y, Xiao Q, et al. Integrative analysis of reference epigenomes in 20 rice varieties.GSE142570. Gene Expression Omnibus. 2020. https://www.ncbi.nlm.nih.gov/geo/query/acc.cgi?acc=GSE142570. 10.1038/s41467-020-16457-5 PMC7253419 32461553

[73] Lu Z, Marand AP, Ricci WA, Ethridge CL, Zhang X, RJ S. The prevalence, evolution and chromatin signatures of plant regulatory elements.GSE128434. Gene Expression Omnibus. 2019. https://www.ncbi.nlm.nih.gov/geo/query/acc.cgi?acc=GSE128434. 10.1038/s41477-019-0548-z 31740772

[74] Tan F, Zhou C, Zhou Q, Zhou S, Yang W, Zhao Y, Li G, DX Z. Analysis of chromatin regulators reveals specific features of rice DNA methylation pathways.GSE81436. Gene Expression Omnibus. 2016. https://www.ncbi.nlm.nih.gov/geo/query/acc.cgi?acc=GSE81436. 10.1104/pp.16.00393 PMC4936571 27208249

[75] Tan FQ, Wang W, Li J, Lu Y, Zhu B, Hu F, Li Q, Zhao Y, DX Z. A coiled-coil protein associates Polycomb repressive complex 2 with KNOX/BELL transcription factors to maintain silencing of cell differentiation-promoting genes in the shoot apex.PRJNA758693. Gene Expression Omnibus. 2021. https://www.ncbi.nlm.nih.gov/bioproject/PRJNA758693. 10.1093/plcell/koac133 PMC9338815 35512211

