# Supplementary material for: A DNA adenine demethylase impairs PRC2-mediated repression of genes marked by a specific chromatin signature
Source: Genome Biol. 2023 Aug 30;24:198. doi: 10.1186/s13059-023-03042-4 (PMC10469495; doi:10.1186/s13059-023-03042-4)
Supplement: Supplementary file 1 — Additional file 1: Fig. S1. Production and analysis of ALKBH1 mutants and Overexpression lines. Fig. S2. A set of genes display a specific chromatin signature in the rice genome. Fig. S3. Production and test of ALKBH1 antibody for ChIP-seq. Fig. S4. Histone modifications levels in ALKBH1 mutant and over-expression plants. Fig. S5. Tests of ALKBH1 H3K27me3 demethylase activity. Fig. S6. ALKBH1 targets to 6mA-methylated R-loop containing genes with euchromatin features to reduced 6mA and H3K27me3 levels. [file 13059_2023_3042_MOESM1_ESM.pptx]

## Slide 1
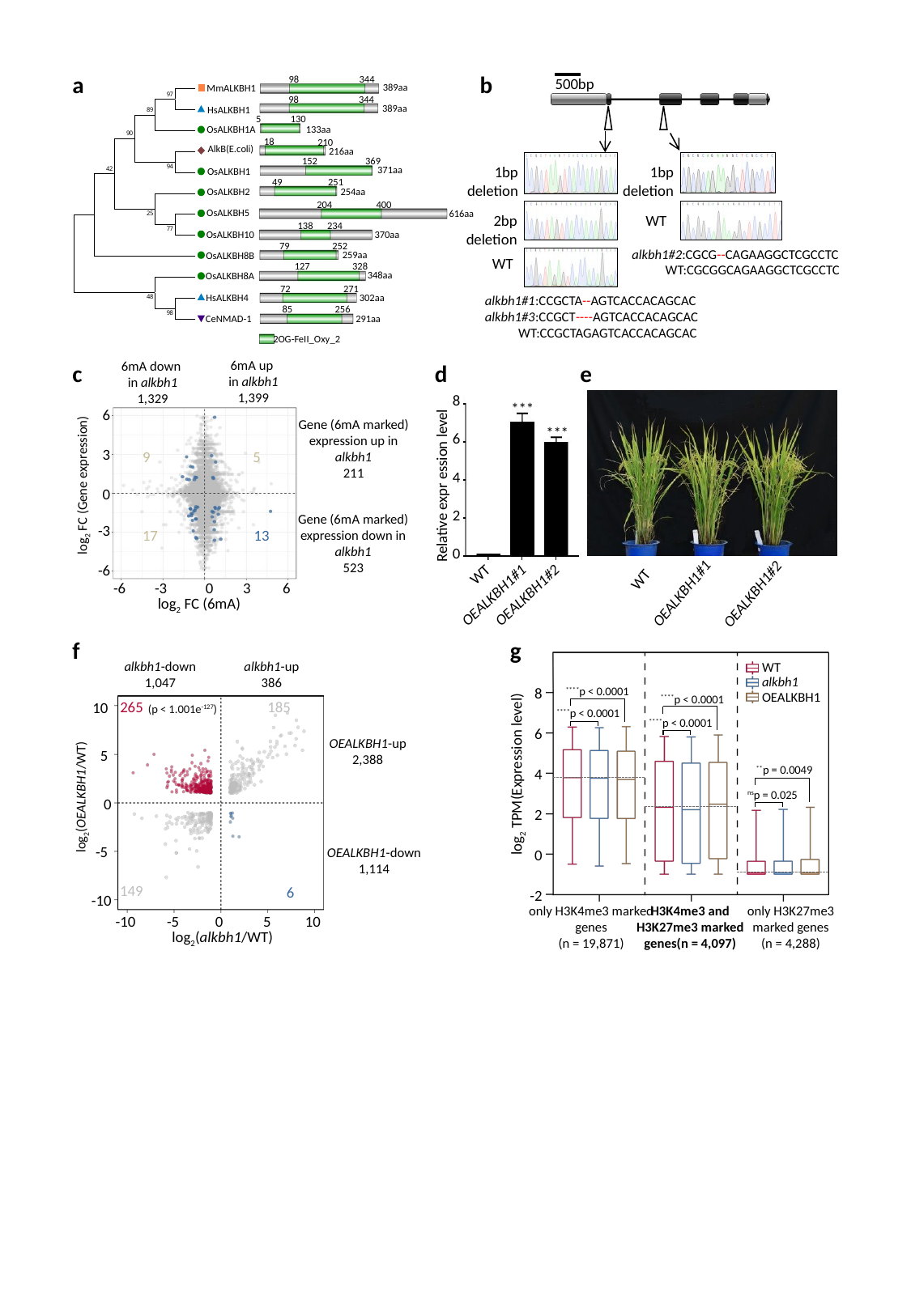

a
b
98
344
389aa
344
98
389aa
5
130
133aa
18
210
216aa
369
152
371aa
49
251
254aa
400
204
616aa
138
234
370aa
79
252
259aa
127
348aa
328
72
271
302aa
85
256
291aa
2OG-FeII_Oxy_2
500bp
1bp deletion
1bp deletion
WT
2bp deletion
alkbh1#2:CGCG--CAGAAGGCTCGCCTC
 WT:CGCGGCAGAAGGCTCGCCTC
WT
alkbh1#1:CCGCTA--AGTCACCACAGCAC
alkbh1#3:CCGCT----AGTCACCACAGCAC
 WT:CCGCTAGAGTCACCACAGCAC
MmALKBH1
HsALKBH1
OsALKBH1A
AlkB(E.coli)
OsALKBH1
OsALKBH2
OsALKBH5
OsALKBH10
OsALKBH8B
OsALKBH8A
HsALKBH4
CeNMAD-1
6mA up
 in alkbh1
 1,399
6mA down
 in alkbh1
 1,329
Gene (6mA marked) expression up in alkbh1
211
Gene (6mA marked) expression down in alkbh1
523
6
3
0
-3
-6
9
5
log2 FC (Gene expression)
17
13
-6
-3
0
3
6
log2 FC (6mA)
c
d
e
8
***
***
6
Relative expr ession level
4
2
0
WT
OEALKBH1#1
OEALKBH1#2
WT
OEALKBH1#1
OEALKBH1#2
f
g
alkbh1-down
1,047
alkbh1-up
386
265
185
10
OEALKBH1-up
2,388
5
log2(OEALKBH1/WT)
0
-5
OEALKBH1-down
1,114
149
6
-10
-10
-5
0
5
10
log2(alkbh1/WT)
(p < 1.001e-127)
WT
alkbh1
OEALKBH1
8
6
4
2
0
-2
****p < 0.0001
****p < 0.0001
****p < 0.0001
****p < 0.0001
log2 TPM(Expression level)
**p = 0.0049
nsp = 0.025
only H3K4me3 marked genes
(n = 19,871)
H3K4me3 and H3K27me3 marked genes(n = 4,097)
only H3K27me3 marked genes
(n = 4,288)

## Slide 2
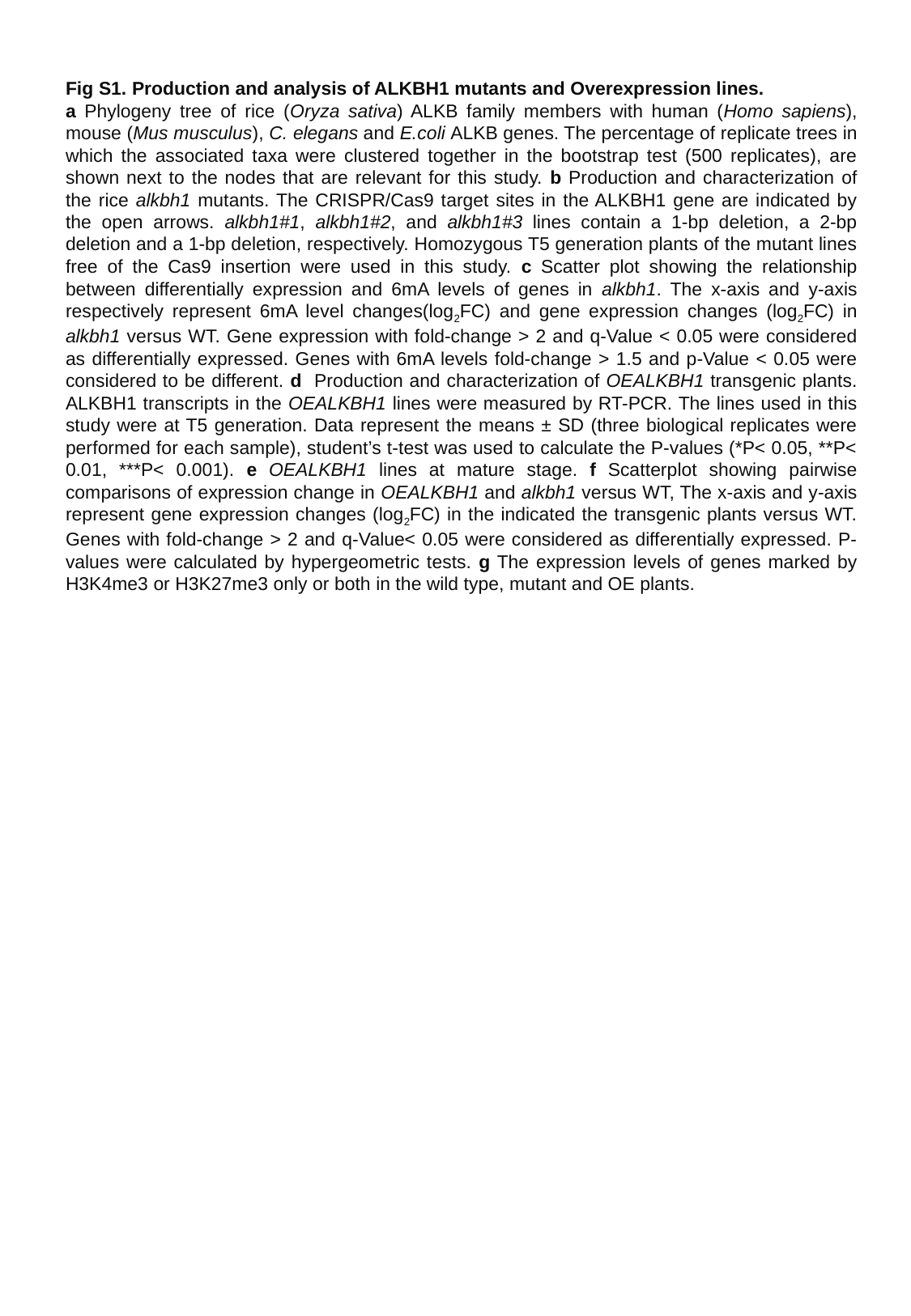

Fig S1. Production and analysis of ALKBH1 mutants and Overexpression lines.
a Phylogeny tree of rice (Oryza sativa) ALKB family members with human (Homo sapiens), mouse (Mus musculus), C. elegans and E.coli ALKB genes. The percentage of replicate trees in which the associated taxa were clustered together in the bootstrap test (500 replicates), are shown next to the nodes that are relevant for this study. b Production and characterization of the rice alkbh1 mutants. The CRISPR/Cas9 target sites in the ALKBH1 gene are indicated by the open arrows. alkbh1#1, alkbh1#2, and alkbh1#3 lines contain a 1-bp deletion, a 2-bp deletion and a 1-bp deletion, respectively. Homozygous T5 generation plants of the mutant lines free of the Cas9 insertion were used in this study. c Scatter plot showing the relationship between differentially expression and 6mA levels of genes in alkbh1. The x-axis and y-axis respectively represent 6mA level changes(log2FC) and gene expression changes (log2FC) in alkbh1 versus WT. Gene expression with fold-change > 2 and q-Value < 0.05 were considered as differentially expressed. Genes with 6mA levels fold-change > 1.5 and p-Value < 0.05 were considered to be different. d Production and characterization of OEALKBH1 transgenic plants. ALKBH1 transcripts in the OEALKBH1 lines were measured by RT-PCR. The lines used in this study were at T5 generation. Data represent the means ± SD (three biological replicates were performed for each sample), student’s t-test was used to calculate the P-values (*P< 0.05, **P< 0.01, ***P< 0.001). e OEALKBH1 lines at mature stage. f Scatterplot showing pairwise comparisons of expression change in OEALKBH1 and alkbh1 versus WT, The x-axis and y-axis represent gene expression changes (log2FC) in the indicated the transgenic plants versus WT. Genes with fold-change > 2 and q-Value< 0.05 were considered as differentially expressed. P-values were calculated by hypergeometric tests. g The expression levels of genes marked by H3K4me3 or H3K27me3 only or both in the wild type, mutant and OE plants.

## Slide 3
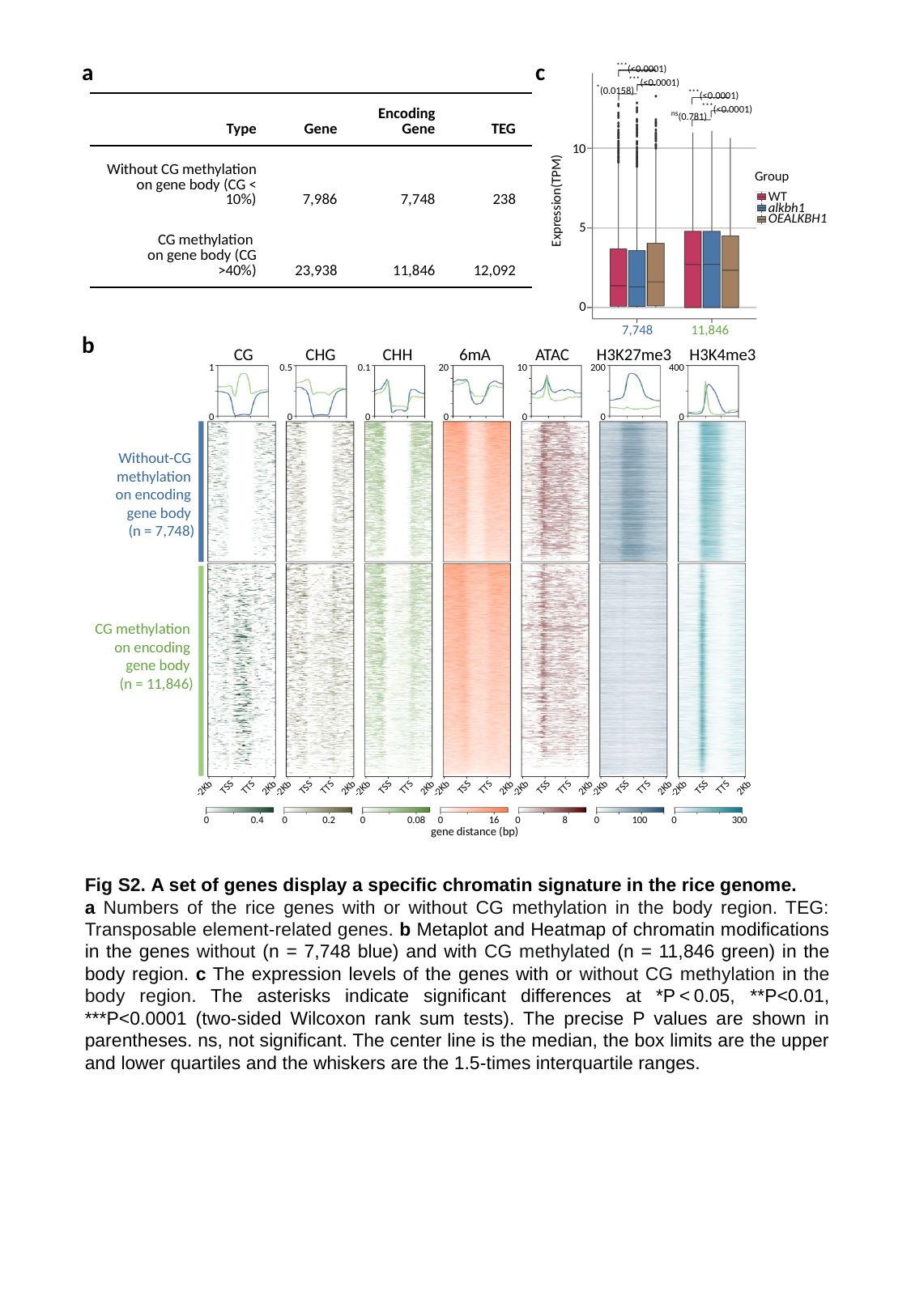

a
c
***(<0.0001)
***(<0.0001)
*(0.0158)
***(<0.0001)
| Type | Gene | Encoding Gene | TEG |
| --- | --- | --- | --- |
| Without CG methylation on gene body (CG < 10%) | 7,986 | 7,748 | 238 |
| CG methylation on gene body (CG >40%) | 23,938 | 11,846 | 12,092 |
***(<0.0001)
ns(0.781)
10
Group
WT
Expression(TPM)
alkbh1
OEALKBH1
5
0
7,748
11,846
b
CG
CHG
CHH
6mA
ATAC
H3K27me3
H3K4me3
1
0.5
0.1
20
10
200
400
0
0
0
0
0
0
0
Without-CG
methylation
on encoding
gene body
(n = 7,748)
CG methylation
on encoding
gene body
(n = 11,846)
-2Kb
TSS
TTS
2Kb
-2Kb
TSS
TTS
2Kb
-2Kb
TSS
TTS
2Kb
-2Kb
TSS
TTS
2Kb
-2Kb
TSS
TTS
2Kb
-2Kb
TSS
TTS
2Kb
-2Kb
TSS
TTS
2Kb
0
0.4
0
0.2
0
0.08
0
16
0
8
0
100
0
300
gene distance (bp)
Fig S2. A set of genes display a specific chromatin signature in the rice genome.
a Numbers of the rice genes with or without CG methylation in the body region. TEG: Transposable element-related genes. b Metaplot and Heatmap of chromatin modifications in the genes without (n = 7,748 blue) and with CG methylated (n = 11,846 green) in the body region. c The expression levels of the genes with or without CG methylation in the body region. The asterisks indicate significant differences at *P < 0.05, **P<0.01, ***P<0.0001 (two-sided Wilcoxon rank sum tests). The precise P values are shown in parentheses. ns, not significant. The center line is the median, the box limits are the upper and lower quartiles and the whiskers are the 1.5-times interquartile ranges.

## Slide 4
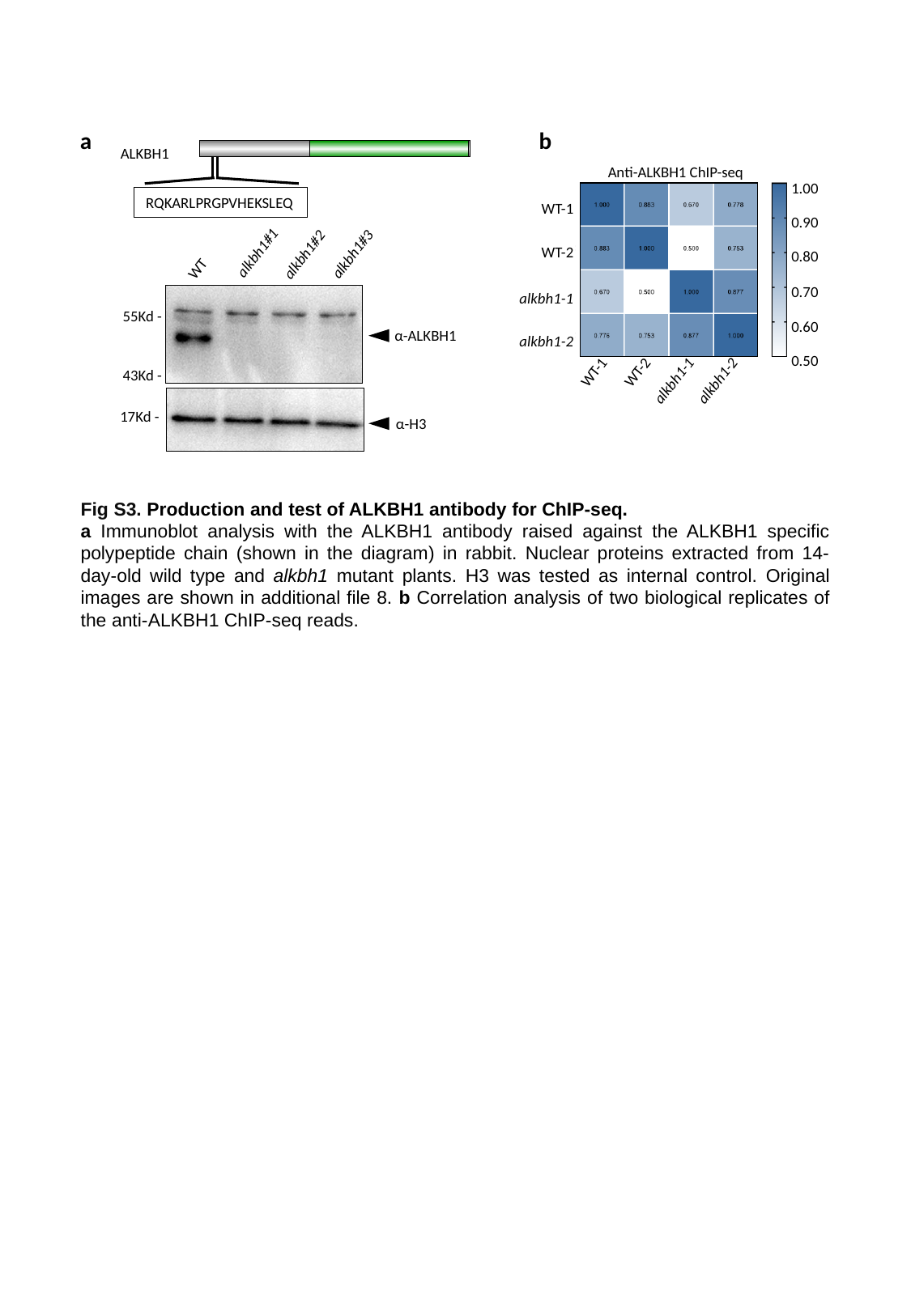

a
b
ALKBH1
RQKARLPRGPVHEKSLEQ
Anti-ALKBH1 ChIP-seq
1.00
0.90
0.80
0.70
0.60
0.50
WT-1
WT-2
alkbh1-1
alkbh1-2
WT-1
WT-2
alkbh1-1
alkbh1-2
alkbh1#1
alkbh1#2
alkbh1#3
WT
55Kd -
α-ALKBH1
43Kd -
17Kd -
α-H3
Fig S3. Production and test of ALKBH1 antibody for ChIP-seq.
a Immunoblot analysis with the ALKBH1 antibody raised against the ALKBH1 specific polypeptide chain (shown in the diagram) in rabbit. Nuclear proteins extracted from 14-day-old wild type and alkbh1 mutant plants. H3 was tested as internal control. Original images are shown in additional file 8. b Correlation analysis of two biological replicates of the anti-ALKBH1 ChIP-seq reads.

## Slide 5
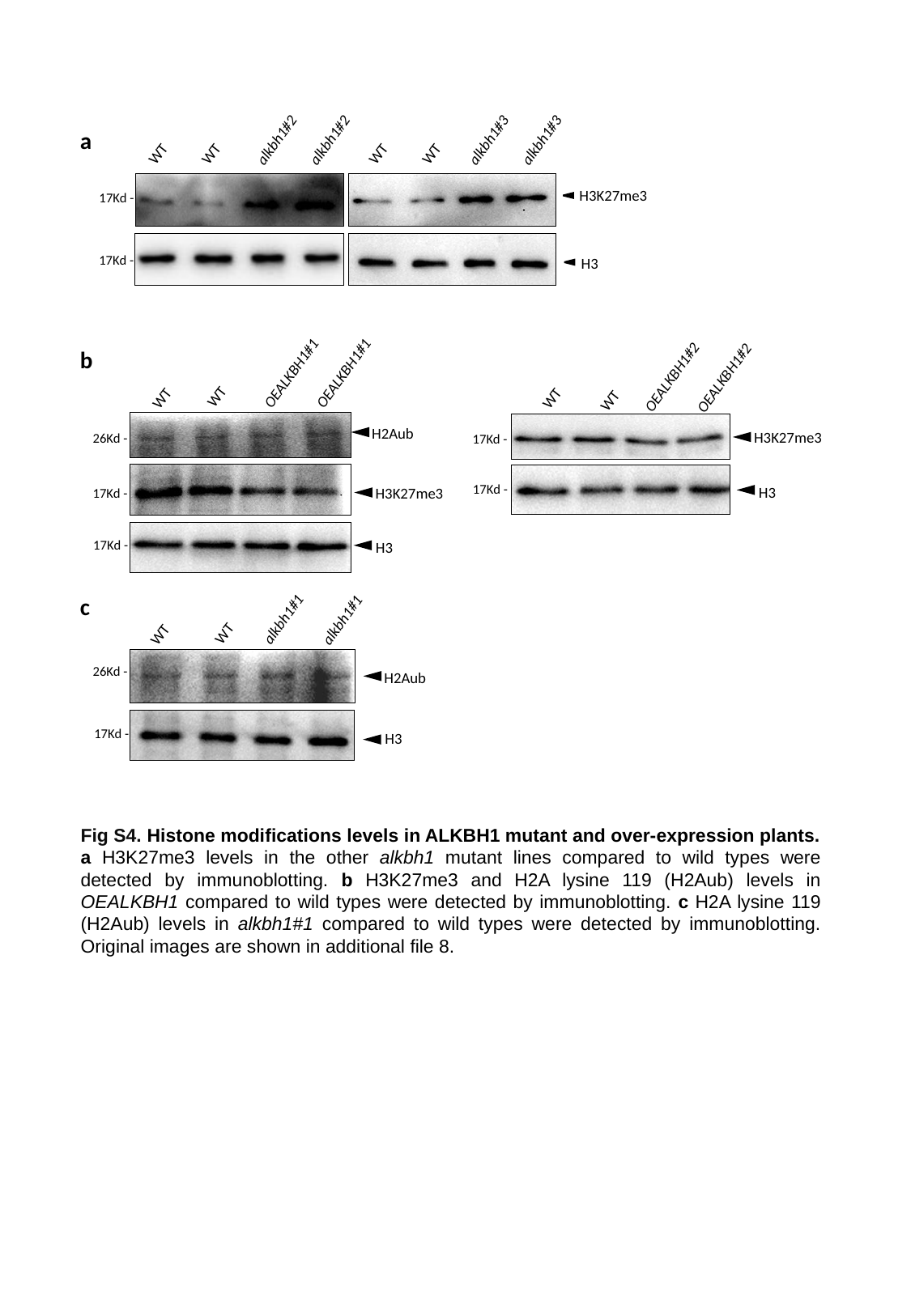

alkbh1#2
alkbh1#2
WT
WT
alkbh1#3
alkbh1#3
WT
WT
H3K27me3
H3
17Kd -
17Kd -
a
OEALKBH1#2
OEALKBH1#2
WT
WT
H3K27me3
17Kd -
17Kd -
H3
OEALKBH1#1
OEALKBH1#1
WT
WT
H2Aub
26Kd -
H3K27me3
17Kd -
17Kd -
H3
b
alkbh1#1
alkbh1#1
WT
WT
H2Aub
H3
26Kd -
17Kd -
c
Fig S4. Histone modifications levels in ALKBH1 mutant and over-expression plants.
a H3K27me3 levels in the other alkbh1 mutant lines compared to wild types were detected by immunoblotting. b H3K27me3 and H2A lysine 119 (H2Aub) levels in OEALKBH1 compared to wild types were detected by immunoblotting. c H2A lysine 119 (H2Aub) levels in alkbh1#1 compared to wild types were detected by immunoblotting. Original images are shown in additional file 8.

## Slide 6
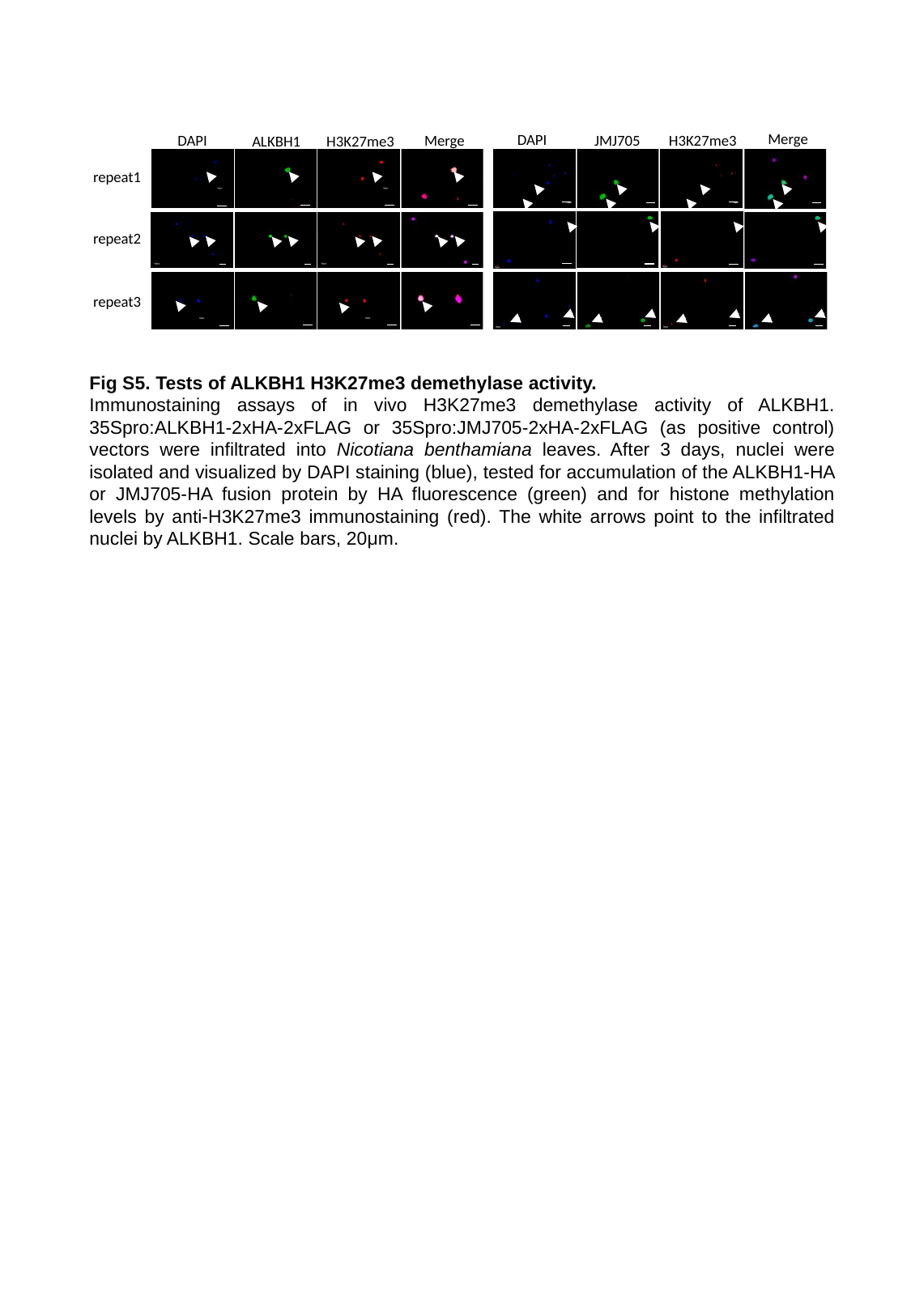

Merge
DAPI
JMJ705
H3K27me3
Merge
DAPI
ALKBH1
H3K27me3
repeat1
repeat2
repeat3
Fig S5. Tests of ALKBH1 H3K27me3 demethylase activity.
Immunostaining assays of in vivo H3K27me3 demethylase activity of ALKBH1. 35Spro:ALKBH1-2xHA-2xFLAG or 35Spro:JMJ705-2xHA-2xFLAG (as positive control) vectors were infiltrated into Nicotiana benthamiana leaves. After 3 days, nuclei were isolated and visualized by DAPI staining (blue), tested for accumulation of the ALKBH1-HA or JMJ705-HA fusion protein by HA fluorescence (green) and for histone methylation levels by anti-H3K27me3 immunostaining (red). The white arrows point to the infiltrated nuclei by ALKBH1. Scale bars, 20μm.

## Slide 7
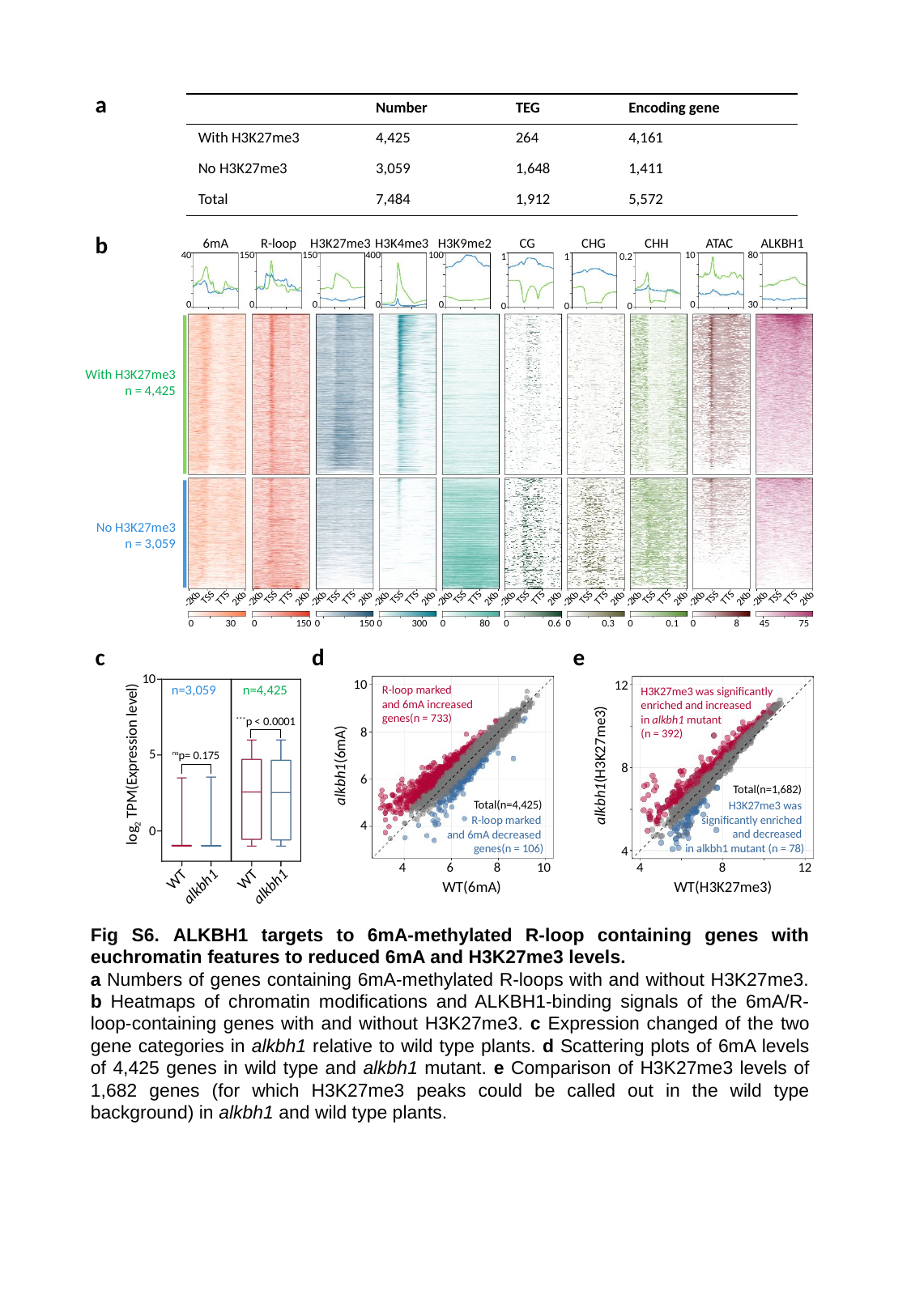

a
| | Number | TEG | Encoding gene |
| --- | --- | --- | --- |
| With H3K27me3 | 4,425 | 264 | 4,161 |
| No H3K27me3 | 3,059 | 1,648 | 1,411 |
| Total | 7,484 | 1,912 | 5,572 |
b
6mA
R-loop
H3K27me3
H3K4me3
H3K9me2
CG
CHG
CHH
ATAC
ALKBH1
40
150
150
400
100
10
80
1
1
0.2
0
0
0
0
0
0
0
0
0
30
With H3K27me3
n = 4,425
No H3K27me3
n = 3,059
TTS
2Kb
TSS
-2Kb
TTS
2Kb
TSS
-2Kb
TTS
2Kb
TSS
-2Kb
TTS
2Kb
TSS
-2Kb
TTS
2Kb
TSS
-2Kb
TTS
2Kb
TSS
-2Kb
TTS
2Kb
TSS
-2Kb
TTS
2Kb
TSS
-2Kb
TTS
2Kb
TSS
-2Kb
TTS
2Kb
TSS
-2Kb
0
30
0
150
0
150
0
300
0
80
0
0.6
0
0.3
0
0.1
0
8
45
75
c
d
e
10
n=3,059
n=4,425
***p < 0.0001
5
nsp= 0.175
log2 TPM(Expression level)
0
WT
alkbh1
WT
alkbh1
10
R-loop marked
and 6mA increased
genes(n = 733)
8
alkbh1(6mA)
6
Total(n=4,425)
R-loop marked
and 6mA decreased
genes(n = 106)
4
4
6
8
10
WT(6mA)
12
H3K27me3 was significantly
enriched and increased
in alkbh1 mutant
(n = 392)
alkbh1(H3K27me3)
8
Total(n=1,682)
H3K27me3 was
significantly enriched
and decreased
in alkbh1 mutant (n = 78)
4
4
8
12
WT(H3K27me3)
Fig S6. ALKBH1 targets to 6mA-methylated R-loop containing genes with euchromatin features to reduced 6mA and H3K27me3 levels.
a Numbers of genes containing 6mA-methylated R-loops with and without H3K27me3. b Heatmaps of chromatin modifications and ALKBH1-binding signals of the 6mA/R-loop-containing genes with and without H3K27me3. c Expression changed of the two gene categories in alkbh1 relative to wild type plants. d Scattering plots of 6mA levels of 4,425 genes in wild type and alkbh1 mutant. e Comparison of H3K27me3 levels of 1,682 genes (for which H3K27me3 peaks could be called out in the wild type background) in alkbh1 and wild type plants.
