# Supplementary material for: A DNA adenine demethylase impairs PRC2-mediated repression of genes marked by a specific chromatin signature
Source: Genome Biol. 2023 Aug 30;24:198. doi: 10.1186/s13059-023-03042-4 (PMC10469495; doi:10.1186/s13059-023-03042-4)
Supplement: Supplementary file 2 — Additional file 2: Table S1. 6mA DIP-seq, RNA-seq and ChIP-seq data alignment summary. [file 13059_2023_3042_MOESM2_ESM.pptx]

## Slide 1
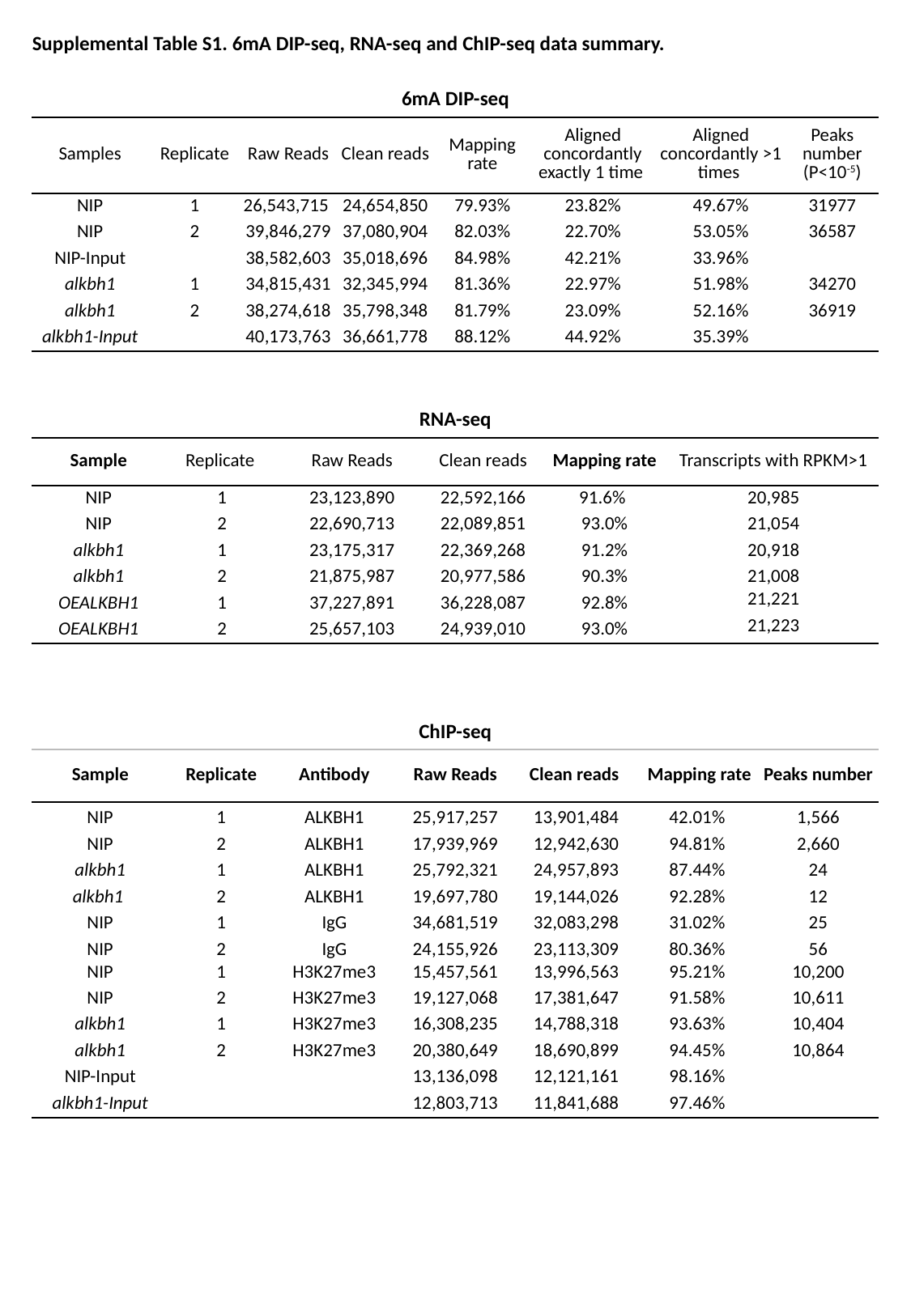

Supplemental Table S1. 6mA DIP-seq, RNA-seq and ChIP-seq data summary.
6mA DIP-seq
| Samples | Replicate | Raw Reads | Clean reads | Mapping rate | Aligned concordantly exactly 1 time | Aligned concordantly >1 times | Peaks number (P<10-5) |
| --- | --- | --- | --- | --- | --- | --- | --- |
| NIP | 1 | 26,543,715 | 24,654,850 | 79.93% | 23.82% | 49.67% | 31977 |
| NIP | 2 | 39,846,279 | 37,080,904 | 82.03% | 22.70% | 53.05% | 36587 |
| NIP-Input | | 38,582,603 | 35,018,696 | 84.98% | 42.21% | 33.96% | |
| alkbh1 | 1 | 34,815,431 | 32,345,994 | 81.36% | 22.97% | 51.98% | 34270 |
| alkbh1 | 2 | 38,274,618 | 35,798,348 | 81.79% | 23.09% | 52.16% | 36919 |
| alkbh1-Input | | 40,173,763 | 36,661,778 | 88.12% | 44.92% | 35.39% | |
RNA-seq
| Sample | Replicate | Raw Reads | Clean reads | Mapping rate | Transcripts with RPKM>1 |
| --- | --- | --- | --- | --- | --- |
| NIP | 1 | 23,123,890 | 22,592,166 | 91.6% | 20,985 |
| NIP | 2 | 22,690,713 | 22,089,851 | 93.0% | 21,054 |
| alkbh1 | 1 | 23,175,317 | 22,369,268 | 91.2% | 20,918 |
| alkbh1 | 2 | 21,875,987 | 20,977,586 | 90.3% | 21,008 |
| OEALKBH1 | 1 | 37,227,891 | 36,228,087 | 92.8% | 21,221 |
| OEALKBH1 | 2 | 25,657,103 | 24,939,010 | 93.0% | 21,223 |
ChIP-seq
| Sample | Replicate | Antibody | Raw Reads | Clean reads | Mapping rate | Peaks number |
| --- | --- | --- | --- | --- | --- | --- |
| NIP | 1 | ALKBH1 | 25,917,257 | 13,901,484 | 42.01% | 1,566 |
| NIP | 2 | ALKBH1 | 17,939,969 | 12,942,630 | 94.81% | 2,660 |
| alkbh1 | 1 | ALKBH1 | 25,792,321 | 24,957,893 | 87.44% | 24 |
| alkbh1 | 2 | ALKBH1 | 19,697,780 | 19,144,026 | 92.28% | 12 |
| NIP | 1 | IgG | 34,681,519 | 32,083,298 | 31.02% | 25 |
| NIP | 2 | IgG | 24,155,926 | 23,113,309 | 80.36% | 56 |
| NIP | 1 | H3K27me3 | 15,457,561 | 13,996,563 | 95.21% | 10,200 |
| NIP | 2 | H3K27me3 | 19,127,068 | 17,381,647 | 91.58% | 10,611 |
| alkbh1 | 1 | H3K27me3 | 16,308,235 | 14,788,318 | 93.63% | 10,404 |
| alkbh1 | 2 | H3K27me3 | 20,380,649 | 18,690,899 | 94.45% | 10,864 |
| NIP-Input | | | 13,136,098 | 12,121,161 | 98.16% | |
| alkbh1-Input | | | 12,803,713 | 11,841,688 | 97.46% | |
