# Supplementary material for: A DNA adenine demethylase impairs PRC2-mediated repression of genes marked by a specific chromatin signature
Source: Genome Biol. 2023 Aug 30;24:198. doi: 10.1186/s13059-023-03042-4 (PMC10469495; doi:10.1186/s13059-023-03042-4)
Supplement: Supplementary file 5 — Additional file 5: Table S4. The increases of H3K27me3, expression level downregulated in alkbh1 and ALKBH1 binding genes. [file 13059_2023_3042_MOESM5_ESM.docx]

**The increases of H3K27me3, expression level downregulated in *alkbh1* and ALKBH1 binding genes**

| **Locus (TIGR)** | **name** | ***alkbh1*-mRNA-level** | ***alkbh1*-H3K27me3-level** | **ALKBH1 binding** | **Function** |
| --- | --- | --- | --- | --- | --- |
| LOC_Os04g23550 | RERJ1/OsbHLH6 | Down | Up | Yes | Pathogen stress (Meng et al., 2020) |
| LOC_Os01g72530 | OsMSR2 | Down | Up | Yes | Salt and drought stress(Xu et al., 2011) |
| LOC_Os06g09310 |  | Down | Up | Yes | Unknown |
| LOC_Os08g07660 | OsPep3 | Down | Up | Yes | Pathogen stress(Shen et al., 2022) |
| LOC_Os03g20090 | OsMYB2 | Down | Up | Yes | osmotic stress(Yang et al., 2012) |
| LOC_Os02g41510 | OsMYB30 | Down | Up | Yes | Pathogen stress(Li et al., 2020),  cold stress(Lv et al., 2017) |
| LOC_Os03g45860 | OsSAUR17 | Down | Up | Yes | Unknown |
| LOC_Os03g32230 | ZOS3-12 | Down | Up | Yes | Pathogen stress(Li et al., 2017),  osmotic stress(Zhang et al., 2014) |
| LOC_Os02g08270 |  | Down | Up | Yes | Unknown |
| LOC_Os02g52210 | OsRING-1 | Down | Up | Yes | Unknown |
| LOC_Os02g08440 | OsWRKY71 | Down | Up | Yes | Pathogen stress(Liu et al., 2007),  cold stress(Kim et al., 2016) |
| LOC_Os12g28710 |  | Down | Up | Yes | Unknown |
| LOC_Os06g44010 | OsWRKY28 | Down | Up | Yes | Pathogen stress(Chujo et al., 2013) |
| LOC_Os09g37080 |  | Down | Up | Yes | Unknown |
| LOC_Os06g08460 |  | Down | Up | Yes | Unknown |
| LOC_Os12g05440 | OsCYP94C2b | Down | Up | Yes | Salt stress(Kurotani et al., 2015) |
| LOC_Os06g33970 | OsVQ22 | Down | Up | Yes | Unknown |
| LOC_Os01g46800 | OsWRKY15 | Down | Up | Yes | Unknown |
| LOC_Os02g45780 | DHS | Down | Up | Yes | drought stress(Wang et al., 2018) |
| LOC_Os02g34410 | OsPUB43 | Down | Up | Yes | drought stress(Wu et al., 2022) |
| LOC_Os04g39010 |  | Down | Up | Yes | Unknown |
| LOC_Os05g27730 | OsWRKY53 | Down | Up | Yes | Pathogen stress(Xie et al., 2021)  cold stress(Hao et al., 2022) |
| LOC_Os06g06360 | OsWRKY113 | Down | Up | Yes | Unknown |
| LOC_Os08g34580 | OsTPS1 | Down | Up | Yes | cold stress salt stress and  drought stress(Li et al., 2011) |
| LOC_Os07g12340 | OsNAC3 | Down | Up | Yes | Unknown |
| LOC_Os07g40000 | OsLBD38 | Down | Up | Yes | Nitrogen sensitivity(Zhu et al., 2022) |
| LOC_Os03g21030 |  | Down | Up | Yes | Unknown |
| LOC_Os04g54240 |  | Down | Up | Yes | Unknown |
| LOC_Os02g22160 |  | Down | Up | Yes | Unknown |
| LOC_Os02g48860 |  | Down | Up | Yes | Unknown |
| LOC_Os07g40630 |  | Down | Up | Yes | Unknown |
| LOC_Os03g08320 | OsJAZ11 | Down | Up | Yes | Unknown |
| LOC_Os07g48260 | OsWRKY47 | Down | Up | Yes | drought stress(Raineri et al., 2015) |
| LOC_Os07g06840 |  | Down | Up | Yes | Unknown |
| LOC_Os05g34830 | OsNAC52 | Down | Up | Yes | drought stress(Gao et al., 2009) |

Chujo, T., Miyamoto, K., Shimogawa, T., Shimizu, T., Otake, Y., Yokotani, N., Nishizawa, Y., Shibuya, N., Nojiri, H., Yamane, H.*, et al.* (2013). OsWRKY28, a PAMP-responsive transrepressor, negatively regulates innate immune responses in rice against rice blast fungus. Plant Mol Biol *82*, 23-37.

Gao, F., Xiong, A., Peng, R., Jin, X., Xu, J., Zhu, B., Chen, J., and Yao, Q. (2009). OsNAC52, a rice NAC transcription factor, potentially responds to ABA and confers drought tolerance in transgenic plants. Plant Cell, Tissue and Organ Culture (PCTOC) *100*, 255-262.

Hao, Z., Tian, J., Fang, H., Fang, L., Xu, X., He, F., Li, S., Xie, W., Du, Q., You, X.*, et al.* (2022). A VQ-motif-containing protein fine-tunes rice immunity and growth by a hierarchical regulatory mechanism. Cell Rep *40*, 111235.

Kim, C.-Y., Vo, K.T.X., Nguyen, C.D., Jeong, D.-H., Lee, S.-K., Kumar, M., Kim, S.-R., Park, S.-H., Kim, J.-K., and Jeon, J.-S. (2016). Functional analysis of a cold-responsive rice WRKY gene, OsWRKY71. Plant Biotechnology Reports *10*, 13-23.

Kurotani, K., Hayashi, K., Hatanaka, S., Toda, Y., Ogawa, D., Ichikawa, H., Ishimaru, Y., Tashita, R., Suzuki, T., Ueda, M.*, et al.* (2015). Elevated levels of CYP94 family gene expression alleviate the jasmonate response and enhance salt tolerance in rice. Plant Cell Physiol *56*, 779-789.

Li, H.W., Zang, B.S., Deng, X.W., and Wang, X.P. (2011). Overexpression of the trehalose-6-phosphate synthase gene OsTPS1 enhances abiotic stress tolerance in rice. Planta *234*, 1007-1018.

Li, W., Wang, K., Chern, M., Liu, Y., Zhu, Z., Liu, J., Zhu, X., Yin, J., Ran, L., Xiong, J.*, et al.* (2020). Sclerenchyma cell thickening through enhanced lignification induced by OsMYB30 prevents fungal penetration of rice leaves. New Phytol *226*, 1850-1863.

Li, W., Zhu, Z., Chern, M., Yin, J., Yang, C., Ran, L., Cheng, M., He, M., Wang, K., Wang, J.*, et al.* (2017). A Natural Allele of a Transcription Factor in Rice Confers Broad-Spectrum Blast Resistance. Cell *170*, 114-126 e115.

Liu, X., Bai, X., Wang, X., and Chu, C. (2007). OsWRKY71, a rice transcription factor, is involved in rice defense response. J Plant Physiol *164*, 969-979.

Lv, Y., Yang, M., Hu, D., Yang, Z., Ma, S., Li, X., and Xiong, L. (2017). The OsMYB30 Transcription Factor Suppresses Cold Tolerance by Interacting with a JAZ Protein and Suppressing beta-Amylase Expression. Plant Physiol *173*, 1475-1491.

Meng, F., Yang, C., Cao, J., Chen, H., Pang, J., Zhao, Q., Wang, Z., Qing Fu, Z., and Liu, J. (2020). A bHLH transcription activator regulates defense signaling by nucleo-cytosolic trafficking in rice. J Integr Plant Biol *62*, 1552-1573.

Raineri, J., Wang, S., Peleg, Z., Blumwald, E., and Chan, R.L. (2015). The rice transcription factor OsWRKY47 is a positive regulator of the response to water deficit stress. Plant Mol Biol *88*, 401-413.

Shen, W., Zhang, X., Liu, J., Tao, K., Li, C., Xiao, S., Zhang, W., and Li, J.F. (2022). Plant elicitor peptide signalling confers rice resistance to piercing-sucking insect herbivores and pathogens. Plant Biotechnol J *20*, 991-1005.

Wang, Z., Tian, X., Zhao, Q., Liu, Z., Li, X., Ren, Y., Tang, J., Fang, J., Xu, Q., and Bu, Q. (2018). The E3 Ligase DROUGHT HYPERSENSITIVE Negatively Regulates Cuticular Wax Biosynthesis by Promoting the Degradation of Transcription Factor ROC4 in Rice. Plant Cell *30*, 228-244.

Wu, Q., Liu, Y., Xie, Z., Yu, B., Sun, Y., and Huang, J. (2022). OsNAC016 regulates plant architecture and drought tolerance by interacting with the kinases GSK2 and SAPK8. Plant Physiol *189*, 1296-1313.

Xie, W., Ke, Y., Cao, J., Wang, S., and Yuan, M. (2021). Knock out of transcription factor WRKY53 thickens sclerenchyma cell walls, confers bacterial blight resistance. Plant Physiol *187*, 1746-1761.

Xu, G.Y., Rocha, P.S., Wang, M.L., Xu, M.L., Cui, Y.C., Li, L.Y., Zhu, Y.X., and Xia, X. (2011). A novel rice calmodulin-like gene, OsMSR2, enhances drought and salt tolerance and increases ABA sensitivity in Arabidopsis. Planta *234*, 47-59.

Yang, A., Dai, X., and Zhang, W.H. (2012). A R2R3-type MYB gene, OsMYB2, is involved in salt, cold, and dehydration tolerance in rice. J Exp Bot *63*, 2541-2556.

Zhang, H., Liu, Y., Wen, F., Yao, D., Wang, L., Guo, J., Ni, L., Zhang, A., Tan, M., and Jiang, M. (2014). A novel rice C2H2-type zinc finger protein, ZFP36, is a key player involved in abscisic acid-induced antioxidant defence and oxidative stress tolerance in rice. J Exp Bot *65*, 5795-5809.

Zhu, X., Wang, D., Xie, L., Zhou, T., Zhao, J., Zhang, Q., Yang, M., Wu, W., and Lian, X. (2022). Rice transcription factors OsLBD37/38/39 regulate nitrate uptake by repressing OsNRT2.1/2.2/2.3 under high-nitrogen conditions. The Crop Journal *10*, 1623-1632.
