# Supplementary material for: A DNA adenine demethylase impairs PRC2-mediated repression of genes marked by a specific chromatin signature
Source: Genome Biol. 2023 Aug 30;24:198. doi: 10.1186/s13059-023-03042-4 (PMC10469495; doi:10.1186/s13059-023-03042-4)

Uncropped western blotting analysis

Uncropped western blotting and gel for Fig. 4a


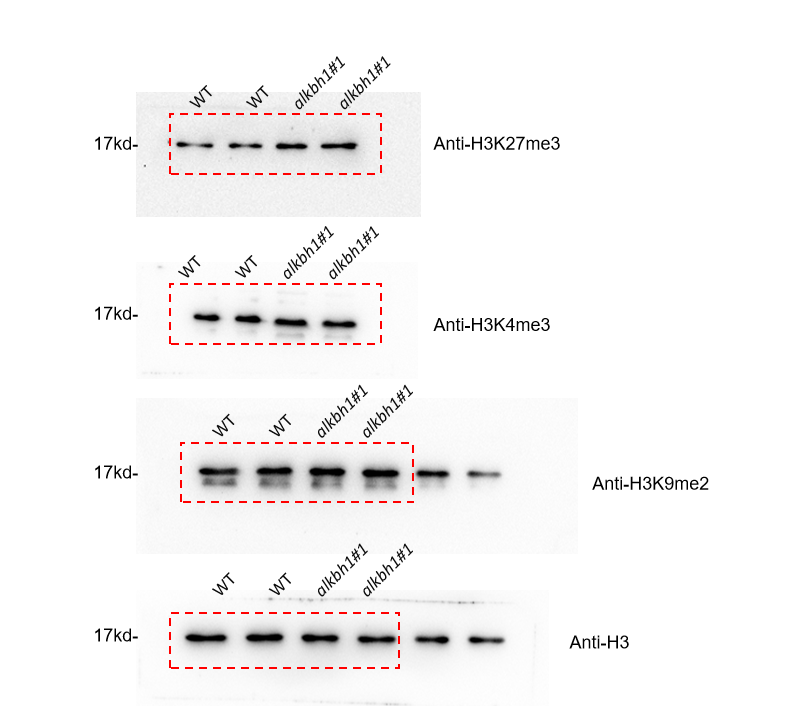


Uncropped western blotting and gel for Fig. s3a


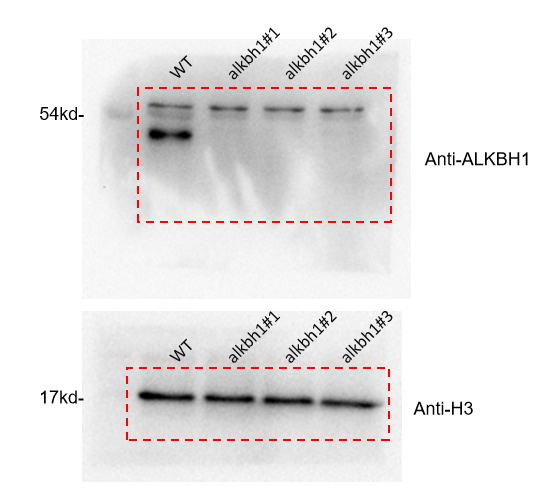


Uncropped western blotting and gel for Fig. s4


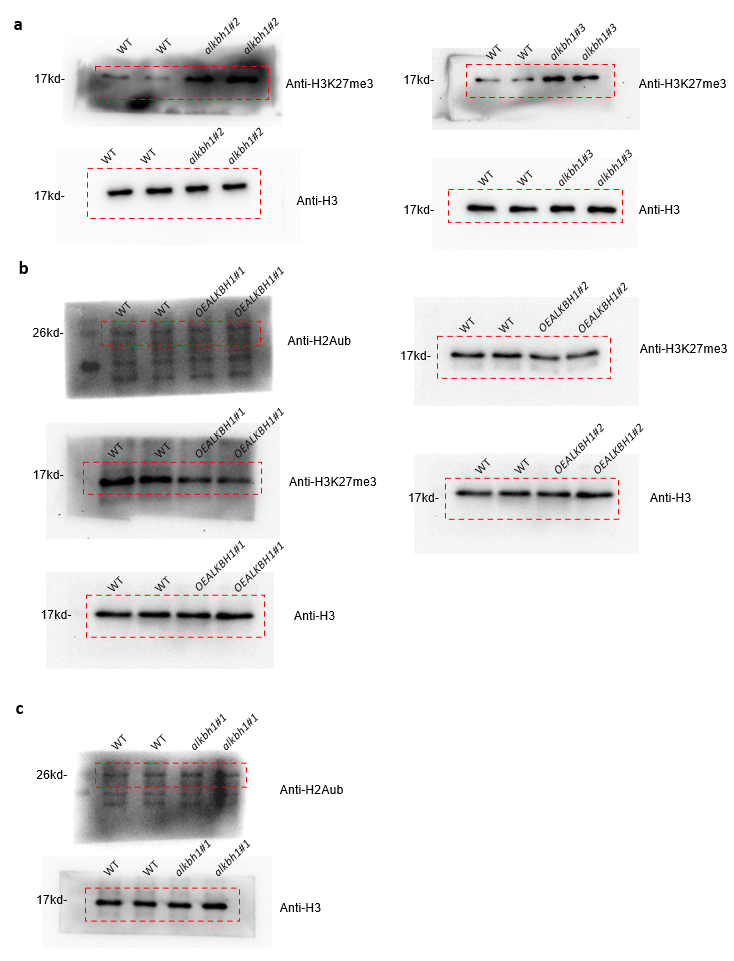

Supplement: Supplementary file 8 — Additional file 8. Uncropped images for the blots. [file 13059_2023_3042_MOESM8_ESM.docx]
