# Supplementary material for: A DNA adenine demethylase impairs PRC2-mediated repression of genes marked by a specific chromatin signature
Source: Genome Biol. 2023 Aug 30;24:198. doi: 10.1186/s13059-023-03042-4 (PMC10469495; doi:10.1186/s13059-023-03042-4)
Supplement: Supplementary file 9 — Additional file 9. Review history. [file 13059_2023_3042_MOESM9_ESM.docx]

Review History

**First round of review**

**Reviewer 1**

**Were you able to assess all statistics in the manuscript, including the appropriateness of statistical tests used?**

Yes, and I have assessed the statistics in my report.

**Comments to author:**

This is an excellent piece of work that reveals the role of rice ALKBH1 in modulating PRC2 function. The manuscript is relatively well written (apart from a few spelling and grammatical errors), the story is logically presented and most of the conclusions are supported by data. The methodology employed is appropriate and with sufficient controls to draw sound conclusions. Clearly, the work presented represents a significant advance on our understanding for DNA adenine demethylase function in plants and I expect it will be of interest to a broad audience. I have however some suggestions that may improve the clarity and quality of the manuscript:

Major point:

- The data suggest that ALKBH1 demethylates 6mA at R-loops but how this is linked to the specific chromatin signatures should be investigated.

Minor points:

p166. Provide quantification of Western Blot shown in Fig 4A.

p176. Revise statement "ALKBH1-binding inhibited H3K27me3 and de-repressed gene expression"

p187. "reprehensive"?

**Reviewer 2**

**Were you able to assess all statistics in the manuscript, including the appropriateness of statistical tests used?**

Yes, and I have assessed the statistics in my report.

**Comments to author:**

The manuscript by Qingxiao Jia et al. describes a novel role for the adenine demethylase ALKBH1 in the moderation of H3K27me3 in rice and proposes its specific function at genes that show enrichment for H3K4me3, H3K27me3 and low CG methylation. The authors perform DIP-seq for m6A and RNA-seq to explore m6A distribution in the context of WT and alkbh1 mutant genetic backgrounds. Based on this data, they suggest the preferential impact of ALKBH1 on genes that carry a specific combination of chromatin features. Due to differences in global level of H3K27me3, they further explore the function of ALKBH1 in context of PRC2 repression and find that ALKBH1 contributes to the reduction of H3K27me3 at stress-responsive genes and ultimately in stress response in rice plants.

The manuscript is very clearly and carefully organised, documented and written. It provides a very valuable resource extending our understanding of m6A demethylases in plants and contributing to the current debate on the impact of m6A in eukaryotes. I agree with the conclusions and implications proposed and demonstrated in the second part of the manuscript, where ALKBH1 is connected to the control of H3K27me3 on subsets of PRC2-target genes. This is an interesting and novel connection that may extend to other model species than rice. I am more critical of the first part that focuses on the m6A and the connection of ALKBH1 activity at genes with specific chromatin signatures, resembling "bivalent" genes. Although the manuscript (logically) starts with looking at m6A, it finally brings evidence that the ALKBH1 activity in counteracting PRC2 repression is independent of its presumed catalytic activity (i.e. demethylation of m6A). It rather demonstrates that ALKBH1 is indirectly (not through m6A) required for the transcriptional control of stress responsive genes, that can be marked by either H3K27me3 or H3K4me3, depending on cell type and condition. ALKBH1 therefore appears as a modulator of H3K27me3 at a limited subset of PRC2 targets, which by itself is interesting but not really related to m6A. In this light, the initial focus on m6A, R-loops etc seems unconnected from the main message of the manuscript, raising two major critical points:

(i) The direct functional connection between ALKBH1 catalytic activity and the gene expression changes is unclear and seems to be independent of m6A deposition. There seems to be no information on the overlap/correlation between m6A (Fig. 1 and related text) and the ALKBH1 binding sites (bound regions - Fig. 3 and related text). Changes in m6A do not seem to directly impact gene expression of genes differentially expressed in ALKBH1 mutant lines (Fig. S1B - lack of association between genes with affected m6A and DEGs in alkbh1). It is not clear how catalytic function of ALKBH1 is connected to the described effect on H3K27me3.

(ii) It is not clear whether ALKBH1 affects genes marked by both H3K27me3 and H3K4me3 or genes marked by either of the two modifications (i.e. facultative heterochromatin genes that are activated under certain conditions)

I think these points need to be clarified or the manuscript needs to be restructured to address these points.

Next, I list particular comments in order as they appear in the text:

1. The rice ALKBH family is said to have 7 members (ref. Zhou et al 2018 - I cannot find this analysis in the cited paper) but only the ALKBH1 has been studied (Zhou et al 2018 and here). Redundancy is suggested on line 91 to explain the lack of effect of the ALKBH1 mutation on m6A. Since the effects observed by manipulation of ALKBH1 levels seem relatively mild, it would be worth to include more insight into the reasoning for selecting ALKBH1.

2. Three CRISPR lines have been generated (alhbh1#1 and alhbh1#3 were generated before - Zhou et al 2018, alhbh1#2 was generated here). alhbh1 was shown before to impact (increase) global 6mA levels 2-3 times (Zhou 2018) but no effect is observed here. It is not clear whether the same mutant line was analysed in both cases (here, alhbh1#1 is said to have been used to generate the genome-wide profiles). Can the authors explain better the comparison between the lines and the seeming discrepancy between the effect on 6mA observed in Zhou et al 2018 and here?

3. Line 96: Please spell-out/define "wR- and cR-loops" Watson DNA strand-related R-loops and Crick DNA strand-related R-loops. What is the implication of the R-loop location to the effect on H3K27me3?

4. It is not immediately clear from the Figure 1 legend whether the DRIP-seq dataset used in the study is the data generated by Fang et al 2019 (ref39)? If so, please specify in the legend to Fig. 1 C-E. It is not clear whether the tissues used for generating the datasets are comparable. Please specify in the methods (used seedling age not specified, here leaves are used but whole seedlings in Fang et al?).

5. Fig. 1D: Please give the p-value of the 184 6mA-down genes overlapping with R-loops - is this significant?

6. Lines 109 - 127, Fig. 2: It is not clear if the chromatin modifications taken for comparison in alkbh1/OEALKBH1 DEGs vs. control genes are taken from WT? The ChIP data is generated in WT seedling in this study - is that correct? If so, please specify in the paragraph. If this is true, than I am not convinced by the conclusions of the correlations between changes in alkbh1/OEALKBH1 gene expression and the chromatin modifications based on the metaplots and heatmaps. An alternative conclusion could be that genes affected by the level of ALKBH1 activity are enriched for H3K27me3-targeted genes, genes without CG body methylation, and genes enriched for H3K4me3 in WT. In particular:

(i) H3K27me3 - the changes (increase) in H3K27me3 in UP and DOWN alkbh1 DEGs may be attributed to the proportion of the H3K27me3-targeted genes in the compared populations and may reflect the proportion of the H3K27me3 targets among the DEGS rather than the level of the modification (seems to be the case based on heatmaps). An alternative interpretation could then be that genes affected in expression change in alkhb1 are enriched for H3K27me3 targets, which is interesting but not the same as suggested.

(ii) The increase of H3K4me3 in alkbh1 down genes may reflect the fact that these genes are more actively transcribed in WT than alkbh1 (expected), which is higher than genome-wide average (control genes). In fact, also the down DEGs seem to have the broadly distributed H3K4me3. H3K4me3 levels should be compared to control population of genes with comparable transcription levels.

(iii) Similar with CG methylation levels: the DEGS are compared to a control population of genes with "averaged expression levels"(lines 111,112) - what does this mean? The reference population should have relatively similar distribution of gene length/transcription….i.e. factors that could influence the level of CG body methylation.

(iv) If the results reflect separate ChIP experiments done using mixed tissues, implications of BOTH H3K27me3 and H3K4me3 at the same loci should be done with care (Line 127). These genes are equally likely be marked by EITHER H3K27me3 or H3K4me3.

7. (Line 140 - 142): The preferential targeting of ALKHB1 to regions with high H3K27me3, high H3K4me3 and low CG methylation is deduced based on the chromatin signature of genes that are changed in the mutant but not impacted by absence of ALKBH1 in terms of m6A. This in my opinion allows conclusions about the misexpressed genes but not about ALKBH1 targeting, and should be separated.

8. Line 125, Fig S1E - How significant is the overlap of 265 genes between down DEGS in alkbh1 (1047) and up DEGs of OEALBH1 (2388)?

9. Please indicate significance of the overlaps of ALKBH1-binding sites and DEGs given in Lines 150-152.

10. Please unify the use of "WT" and "NIP" in the figures.

11. Please unify the use of TTS and TES in the text and the figures.

12. Fig. 5B - Three primers pairs (genomic regions) are shown to have been tested, but it is not clear form the rest of the figure how this data is integrated into the images

13. Fig5D vs E - The ChIP in Fig. 5D is shown including negative controls and IgG but not so in Fig. 5E. Can you please expand on the figure (either main figure or supplement) to be able to judge on the quality of the ChIP? How is normalization done ("relative H3K27me3 level") and what do the error bars indicate in Fig 5 C-E?

14. Lines 207 - 208, Fig.6A: the EMF2 binding around TTS is also seen in the onlyEMF2b genes (although the general enrichment is lower). It is not very clear to me how the preferentaial association of ALKBH1 with H3K27me3-dopositing PRC2 is meant/explained?

15. Line 21 - 212, Fig. 6B: the results seem counter-intuitive: genes bound by EMF2b and ALKBH1 have a higher level of H3K27me3 (Fig6B) than EMF2b-only) but ALKBH1 is hypothesized to prevent the deposition?

**Authors Response**

**Point-by-point responses to the reviewers’ comments:**

Reviewer #1:

This is an excellent piece of work that reveals the role of rice ALKBH1 in modulating PRC2 function. The manuscript is relatively well written (apart from a few spelling and grammatical errors), the story is logically presented and most of the conclusions are supported by data. The methodology employed is appropriate and with sufficient controls to draw sound conclusions. Clearly, the work presented represents a significant advance on our understanding for DNA adenine demethylase function in plants and I expect it will be of interest to a broad audience. I have however some suggestions that may improve the clarity and quality of the manuscript:

Major point:

- The data suggest that ALKBH1 demethylates 6mA at R-loops but how this is linked to the specific chromatin signatures should be investigated.

*Reply: Many thanks for point. We have analyzed the genes containing 6mA-methylated R-loops. The data are shown in Additional file 1: Fig. S6, described and discussed in the MS text (lines 238 - 252, 286 - 289):*

*The analysis supported the hypothesis that ALKBH1 preferentially targeted R-loops within euchromatin to control 6mA and H3K27me3 levels.*

Minor points:

p166. Provide quantification of Western Blot shown in Fig 4A.

*Reply: Thank you for your suggestion. Quantifications are added in Fig. 4a.*

p176. Revise statement "ALKBH1-binding inhibited H3K27me3 and de-repressed gene expression"

*Reply: We have changed the sentence to "ALKBH1-binding reduced H3K27me3 and stimulated gene expression"* *(line 189).*

p187. "reprehensive"?

*Reply: corrected to “representative” (line 201).*

Reviewer #2:

The manuscript by Qingxiao Jia et al. describes a novel role for the adenine demethylase ALKBH1 in the moderation of H3K27me3 in rice and proposes its specific function at genes that show enrichment for H3K4me3, H3K27me3 and low CG methylation. The authors perform DIP-seq for m6A and RNA-seq to explore m6A distribution in the context of WT and alkbh1 mutant genetic backgrounds. Based on this data, they suggest the preferential impact of ALKBH1 on genes that carry a specific combination of chromatin features. Due to differences in global level of H3K27me3, they further explore the function of ALKBH1 in context of PRC2 repression and find that ALKBH1 contributes to the reduction of H3K27me3 at stress-responsive genes and ultimately in stress response in rice plants.

The manuscript is very clearly and carefully organised, documented and written. It provides a very valuable resource extending our understanding of m6A demethylases in plants and contributing to the current debate on the impact of m6A in eukaryotes. I agree with the conclusions and implications proposed and demonstrated in the second part of the manuscript, where ALKBH1 is connected to the control of H3K27me3 on subsets of PRC2-target genes. This is an interesting and novel connection that may extend to other model species than rice. I am more critical of the first part that focuses on the m6A and the connection of ALKBH1 activity at genes with specific chromatin signatures, resembling "bivalent" genes. Although the manuscript (logically) starts with looking at m6A, it finally brings evidence that the ALKBH1 activity in counteracting PRC2 repression is independent of its presumed catalytic activity (i.e. demethylation of m6A). It rather demonstrates that ALKBH1 is indirectly

(not through m6A) required for the transcriptional control of stress responsive genes, that can be marked by either H3K27me3 or H3K4me3, depending on cell type and condition. ALKBH1 therefore appears as a modulator of H3K27me3 at a limited subset of PRC2 targets, which by itself is interesting but not really related to m6A. In this light, the initial focus on m6A, R-loops etc seems unconnected from the main message of the manuscript, raising two major critical points:

1. The direct functional connection between ALKBH1 catalytic activity and the gene expression changes is unclear and seems to be independent of m6A deposition. There seems to be no information on the overlap/correlation between m6A (Fig. 1 and related text) and the ALKBH1 binding sites (bound regions - Fig. 3 and related text). Changes in m6A do not seem to directly impact gene expression of genes differentially expressed in ALKBH1 mutant lines (Fig. S1B - lack of association between genes with affected m6A and DEGs in alkbh1). It is not clear how catalytic function of ALKBH1 is connected to the described effect on H3K27me3.

*Reply: Thank you for the comment. The point is similar to the comments made by reviewer 1.* *We have analyzed the genes (7,484) that show both 6mA and R-loops (Additional file 1: Fig. S6), described and discussed the data in MS text (lines 238 - 252, 286 - 289):*

*The analysis suggested a link between 6mA, loops and H3K27me3 within euchromatin targeted by ALKBH1.*

2. It is not clear whether ALKBH1 affects genes marked by both H3K27me3 and H3K4me3 or genes marked by either of the two modifications (i.e. facultative heterochromatin genes that are activated under certain conditions)

*Reply: Thanks for the comment. We have separated genes showing H3K27me3 or H3K4me3 only from those with both modifications in wild type background and have analyzed their expression levels in alkbh1 plants (please see Additional file 1: Fig. S1g). The analysis indicated that the alkbh1 mutation and overexpression had more effect on genes showing both H3K27me3 and H3K4me3 than H3K27me3 or H3K4me3 alone (lines 135 - 138).*

Particular comments:

1. The rice ALKBH family is said to have 7 members (ref. Zhou et al 2018 - I cannot find this analysis in the cited paper) but only the ALKBH1 has been studied (Zhou et al 2018 and here). Redundancy is suggested on line 91 to explain the lack of effect of the ALKBH1 mutation on m6A. Since the effects observed by manipulation of ALKBH1 levels seem relatively mild, it would be worth to include more insight into the reasoning for selecting ALKBH1.

*Reply: Sorry for the omission. We have put back the phylogeny in Additional file 1: Fig. S1a.*

2.  Three CRISPR lines have been generated (alhbh1#1 and alhbh1#3 were generated before - Zhou et al 2018, alhbh1#2 was generated here). alhbh1 was shown before to impact (increase) global 6mA levels 2-3 times (Zhou 2018) but no effect is observed here. It is not clear whether the same mutant line was analysed in both cases (here, alhbh1#1 is said to have been used to generate the genome-wide profiles). Can the authors explain better the comparison between the lines and the seeming discrepancy between the effect on 6mA observed in Zhou et al 2018 and here?

*Reply: The discrepancy may be partly due to difference in the methods used. DIP-seq* *recovers part of the genomic regions, whereas MS is more sensitive and comprehensive, but also more prone to errors (such as contamination from mRNA or chloroplastic, mitochondrial, or microbe DNA).*

3. Line 96: Please spell-out/define "wR- and cR-loops" Watson DNA strand-related R-loops and Crick DNA strand-related R-loops. What is the implication of the R-loop location to the effect on H3K27me3?

*Reply: Full names added (lines 100 - 101).*

*R-loop effects on H3K27me3 have been previously shown. We have added relevant reference (Ref 50,51) in Discussion (line 286).*

 4. It is not immediately clear from the Figure 1 legend whether the DRIP-seq dataset used in the study is the data generated by Fang et al 2019 (ref39)? If so, please specify in the legend to Fig. 1 C-E. It is not clear whether the tissues used for generating the datasets are comparable. Please specify in the methods (used seedling age not specified, here leaves are used but whole seedlings in Fang et al?).

*Reply: Ref39 is now added in the figure legend.*

*The rice materials used for sequencing in the experiments were all two-week-old seedlings. We have corrected this in the Methods.*

 5. Fig. 1D: Please give the p-value of the 184 6mA-down genes overlapping with R-loops - is this significant?

*Reply: P-values are added in Fig. 1d.*

6. Lines 109 - 127, Fig. 2: It is not clear if the chromatin modifications taken for comparison in alkbh1/OEALKBH1 DEGs vs. control genes are taken from WT? The ChIP data is generated in WT seedling in this study - is that correct? If so, please specify in the paragraph. If this is true, than I am not convinced by the conclusions of the correlations between changes in alkbh1/OEALKBH1 gene expression and the chromatin modifications based on the metaplots and heatmaps. An alternative conclusion could be that genes affected by the level of ALKBH1 activity are enriched for H3K27me3-targeted genes, genes without CG body methylation, and genes enriched for H3K4me3 in WT. In particular:

(i)	H3K27me3 - the changes (increase) in H3K27me3 in UP and DOWN alkbh1 DEGs may be attributed to the proportion of the H3K27me3-targeted genes in the compared populations and may reflect the proportion of the H3K27me3 targets among the DEGS rather than the level of the modification (seems to be the case based on heatmaps). An alternative interpretation could then be that genes affected in expression change in alkhb1 are enriched for H3K27me3 targets, which is interesting but not the same as suggested.

*Reply: We have changed the interpretation as advised (lines 133 - 134):*

*ALKBH1 was required for the expression of genes enriched for H3K27me3 and/or H3K4me3 but without mCG in the gene body in wild type rice seedling tissues.*

(ii)	The increase of H3K4me3 in alkbh1 down genes may reflect the fact that these genes are more actively transcribed in WT than alkbh1 (expected), which is higher than genome-wide average (control genes). In fact, also the down DEGs seem to have the broadly distributed H3K4me3. H3K4me3 levels should be compared to control population of genes with comparable transcription levels.

*Reply: As advised, we have reselected the control genes with similar expression levels to the down DEGs in the mutant for analysis in Fig. 2. Heatmaps of the DEGs in the mutant and control genes in WT are added to the figure.*

(iii)	Similar with CG methylation levels: the DEGS are compared to a control population of genes with "averaged expression levels"(lines 111,112) - what does this mean? The reference population should have relatively similar distribution of gene length/transcription….i.e. factors that could influence the level of CG body methylation.

*Reply: As advised, we have analyzed CG methylation with control genes of similar lengths (CK2) to that of the DEGs. The results are the same as with CK1 (used previously, please see the Fig below). Since there is no clear difference with CK1 and CK2, we choose not to include the new analysis in the revision.*

(iv)  If the results reflect separate ChIP experiments done using mixed tissues, implications of BOTH H3K27me3 and H3K4me3 at the same loci should be done with care (Line 127). These genes are equally likely be marked by EITHER H3K27me3 or H3K4me3.

*Reply: We have reworded the sentence (lines 133 - 134):*

*“The data indicated that ALKBH1 was required for the expression of genes enriched for H3K27me3 and/or H3K4me3 but without mCG in the gene body in wild type rice seedling tissues.”*

7. (Line 140 – 142): The preferential targeting of ALKHB1 to regions with high H3K27me3, high H3K4me3 and low CG methylation is deduced based on the chromatin signature of genes that are changed in the mutant but not impacted by absence of ALKBH1 in terms of m6A. This in my opinion allows conclusions about the misexpressed genes but not about ALKBH1 targeting, and should be separated.

*Reply: OK, we have changed the statement to (lines 152 – 154) “likely represents a chromatin signature of genes downregulated in alkbh1 mutant and upregulated in OE plants.”*

8. Line 125, Fig S1E - How significant is the overlap of 265 genes between down DEGS in alkbh1 (1047) and up DEGs of OEALBH1 (2388)?

*Reply: P-values are added to Additional file 1: Fig. S1f.*

9. Please indicate significance of the overlaps of ALKBH1-binding sites and DEGs given in Lines 150-152.

*Reply: P-Values added in the text (lines 163 - 164).*

10. Please unify the use of "WT" and "NIP" in the figures.

*Reply: All changed to WT.*

11. Please unify the use of TTS and TES in the text and the figures.

*Reply: All changed to TTS.*

12. Fig. 5B - Three primers pairs (genomic regions) are shown to have been tested, but it is not clear form the rest of the figure how this data is integrated into the images.

*Reply: Primer sets are indicated under each bar for ChIP-PCRs, for RT-PCR, primer sets P2 were used, now indicated in the legend.*

13. Fig5D vs E - The ChIP in Fig. 5D is shown including negative controls and IgG but not so in Fig. 5E. Can you please expand on the figure (either main figure or supplement) to be able to judge on the quality of the ChIP? How is normalization done ("relative H3K27me3 level") and what do the error bars indicate in Fig 5 C-E?

*Reply: Data with IgG were added in Fig. 5e. Normalization was performed using IP/Input.*

*Values are means of three biological replicates and error bars represent -/+ SD, now indicated in the legend.*

14. Lines 207 - 208, Fig.6A: the EMF2 binding around TTS is also seen in the onlyEMF2b genes (although the general enrichment is lower). It is not very clear to me how the preferentaial association of ALKBH1 with H3K27me3-depositing PRC2 is meant/explained?

*Reply: We have changed the statement to (line 222) “suggesting that ALKBH1 may preferentially associate with the loci broadly bound by EMF2b.”*

15. Line 21 - 212, Fig. 6B: the results seem counter-intuitive: genes bound by EMF2b and ALKBH1 have a higher level of H3K27me3 (Fig6B) than EMF2b-only) but ALKBH1 is hypothesized to prevent the deposition?

*Reply: This is good point. This is because a large portion of EMF2b-binding genes have no H3K27me3 in the rice genome (probably those gene are only poised for H3K27me3 (Ref 37)). We have added the explanation in the MS text (lines 224 - 225).*

**Second round of review**

**Reviewer 1**

All my comments have been addressed.

**Reviewer 2**

Thank you for addressing the comments. The authors have largely reflected the comments made. The minor points have been addressed. The addition of analyses depicted in Fig S6 are very valuable and respond to one major comment.
My second major concern related to the possibility to conclude on marking by BOTH H3K27me3 and H3K4me3 still holds - these conclusions are not possible to make with the current data (without having sequential ChIP data or data from separate cell types). In a pool of seedling tissues, the gene category “H3K4me3-marked and H3K27me3-marked genes” (Fig. S1g) may still mean that in some cell types this is a H3K4me3-marked gene and in others H3K27me3-marked. The differences in H3K4me3/H3K27me3 may therefore result from the different proportion of cells that carry either/or modification on the corresponding locus. I therefore think that lines 135-138 are not a very convincing support for the statements in the abstract “However, the ALKBH1 mutation or over-expression mainly affected the expression of genes with a SPECIFIC COMBINATION of chromatin modifications in the body region marked with H3K4me3 and H3K27me3 but depleted of DNA CG methylation.” (line 19). Therefore, I think that the authors should rephrase the conclusions or clearly state that “COMBINATION” of the modifications or their “H3K4me3/H3K27me3 RATIO” (e.g. line 22, line 173) or “BALANCE” (line 25) is meant in the context of complex (mixed) tissue and not on individual loci/genes. This is by no means devaluating the very interesting findings, rather suggesting to avoid formulations that may be misinterpreted by the community, especially since bivalent genes are discussed (lines 73-75).

Minor comments on the modified taxt/figures
- Fig S1g – the number of the “only H3K4me3-marked genes” seems to be badly formatted
- Line 138: it is not clear how the conclusion is reached: do the authors refer to the p-values or the differences of the means (box plots)?

**Authors Response**

**Point-by-point responses to the reviewers’ comments:**

Reviewer #2:

Thank you for addressing the comments. The authors have largely reflected the comments made. The minor points have been addressed. The addition of analyses depicted in Fig S6 are very valuable and respond to one major comment.
My second major concern related to the possibility to conclude on marking by BOTH H3K27me3 and H3K4me3 still holds - these conclusions are not possible to make with the current data (without having sequential ChIP data or data from separate cell types). In a pool of seedling tissues, the gene category “H3K4me3-marked and H3K27me3-marked genes” (Fig. S1g) may still mean that in some cell types this is a H3K4me3-marked gene and in others H3K27me3-marked. The differences in H3K4me3/H3K27me3 may therefore result from the different proportion of cells that carry either/or modification on the corresponding locus. I therefore think that lines 135-138 are not a very convincing support for the statements in the abstract “However, the ALKBH1 mutation or over-expression mainly affected the expression of genes with a SPECIFIC COMBINATION of chromatin modifications in the body region marked with H3K4me3 and H3K27me3 but depleted of DNA CG methylation.” (line 19). Therefore, I think that the authors should rephrase the conclusions or clearly state that “COMBINATION” of the modifications or their “H3K4me3/H3K27me3 RATIO” (e.g. line 22, line 173) or “BALANCE” (line 25) is meant in the context of complex (mixed) tissue and not on individual loci/genes. This is by no means devaluating the very interesting findings, rather suggesting to avoid formulations that may be misinterpreted by the community, especially since bivalent genes are discussed (lines 73-75).

*REPLY: Thanks for the comments. It is now clearly stated that “COMBINATION” of the modifications or their “H3K4me3/H3K27me3 RATIO” (Abstract, line 173, 135-138) or “BALANCE” (Abstract) is meant in the context of complex (mixed) tissue and not on individual loci/genes.*

Minor comments on the modified taxt/figures
- Fig S1g – the number of the “only H3K4me3-marked genes” seems to be badly formatted
*REPLY: The number is reformatted.*
- Line 138: it is not clear how the conclusion is reached: do the authors refer to the p-values or the differences of the means (box plots)?
*REPLY: That was the differences of means. We have made it clear in lines 135-138.*
